# Identification of abnormal neural language networks by reading “brainprints” in patients with brain tumors

**DOI:** 10.1016/j.ynirp.2026.100374

**Published:** 2026-06-20

**Authors:** Pia Ritter, Manuela Christine Michenthaler, Karla Zaar, Kariem Mahdy Ali, Gernot Reishofer, Stefan Wolfsberger, Hannes Deutschmann, Margit Jehna

**Affiliations:** a Department of Radiology, Clinical Division of Neuroradiology, Vascular and Interventional Radiology, Medical University of Graz, Austria; b Department of Neurosurgery, Medical University of Graz, Austria; c Department of Radiology, Medical University of Graz, Austria

**Keywords:** Brain tumors, Connectivity fingerprint, Single-subject analysis, Language network, Functional reorganization, Resting-state fMRI

## Abstract

**Objectives:**

Alterations in neural language networks are common in patients with brain tumors, yet their nature varies substantially across individuals. By reducing data to group-level averages, conventional analyses fail to capture such heterogeneity, obscuring patient-specific information.

**Methods:**

The present study applied a resting-state connectivity fingerprinting approach to characterize language network alterations at the single-subject level, yielding individualized connectivity profiles (“fingerprints”). Fingerprints of 27 right-handed patients with a left-hemisphere brain tumor affecting language-relevant areas were assessed at three time points (preoperative, immediate postoperative and three-month follow-up). Connectivity patterns were compared to a normative reference derived from 30 healthy participants and linked to language performance.

**Results:**

Fingerprints remained temporally stable in healthy individuals. In patients, fingerprints revealed distinct, patient-specific deviations from the typical network structure with highly heterogeneous changes over time. Three main findings emerged: (1) patients with language deficits showed greater deviations from the typical fingerprint than those without deficits; (2) significant associations between larger deviations and poorer language performance were confined to the immediate postoperative phase, likely reflecting surgery- or treatment-related influences or differences in the (mal)adaptivity of reorganization over time; (3) in high-grade glioma, exploratory analyses provided preliminary evidence for an adaptive contribution of the contralesional hemisphere immediately after surgery.

**Conclusions:**

The findings support connectivity fingerprinting as a promising approach for characterizing patient-specific network patterns and monitoring functional reorganization processes relevant to language function at the single-subject level. With continued methodological refinement, this approach holds potential for contributing to more individualized clinical decision-making within the context of personalized medicine.

## Highlights

• Connectivity fingerprints offer a robust measure of brain network (re-)organization.
• Fingerprints revealed patient-specific connectivity patterns in a brain tumor cohort.
• Fingerprinting enables individualized tracking of reorganization processes over time.
• Patient network deviations from healthy fingerprints are linked to language deficits.

## 1 Introduction

Functional neuroimaging methods provide a powerful means to study brain networks by mapping neural activity and interregional connections. In healthy individuals, functional networks display a shared organizational architecture and remain temporally stable across subjects (Akiki and Abdallah, 2019; Finn et al., 2015; Yeo et al., 2011). In contrast, patients with brain tumors frequently exhibit altered connectivity patterns, which may manifest as decreased activation or compensatory upregulation, depending on the tumor's type, grade, and location (Chen et al., 2022; Hart et al., 2019; Jütten et al., 2020; Maesawa et al., 2015). Among the various functional systems in the brain, language relies on a complex and widely distributed network, involving frontal, temporal, and parietal regions (Braga et al., 2020; Fedorenko et al., 2024; Price, 2012). Disruption of critical nodes within the language network can cause measurable impairments, with alterations in functional connectivity commonly linked to tumor- or treatment-related factors (Pasquini et al., 2023; Yuan et al., 2020, 2022). However, the brain has the capacity to undergo functional reorganization, which is generally assumed to serve a compensatory role. In the context of language, redistribution may extend from tumor-affected to perilesional regions or even to remote cortical areas, including the contralateral hemisphere (Deverdun et al., 2020; Duffau, 2014). Such neuroplastic processes may emerge acutely or gradually over time and have been repeatedly associated with language performance (Cargnelutti et al., 2020; Nieberlein et al., 2023). Despite growing interest in neuroplasticity, the exact mechanisms underlying these processes and their clinical implications remain insufficiently understood. Reorganization patterns appear to differ substantially across patients, suggesting that interindividual variability in patients' characteristics plays a key role in determining whether and how the brain adapts to tumor- or treatment-related disruptions (Voets et al., 2019, 2021). Most current scientific understanding of the brain is derived from group-level analyses, which average data across individuals. While these analyses provide valuable insights, they obscure subject-specific variability and require substantial and homogeneous sample sizes, which are often difficult to achieve in clinical populations. Consequently, research relying solely on group statistics is subject to certain constraints in terms of clinical applicability.

Such limitations find resonance within the framework of personalized medicine, which shifts the focus toward patient-specific characteristics and aims to tailor treatment to individuals (Whitcomb, 2012). Building on this rationale, the single-subject connectivity fingerprinting approach introduced by Mars et al. (2016) enables analyses of brain networks at the subject-specific level. A connectivity fingerprint reflects the unique pattern of connectivity in an individual, measured as how one brain region interacts with others. Crucially, the method emphasizes whole-pattern connectivity and thereby allows capturing the broader nature of network disruptions, which rarely affect only a single connection. However, at the same time, it retains the capacity to examine individual connections (Mars et al., 2016; Neubert et al., 2015). Originally developed within cognitive neuroscience, connectivity fingerprinting has demonstrated feasibility in clinical contexts ranging from brain tumor patients (Voets et al., 2019, 2021) to neurodevelopmental disorders (Balsters et al., 2018).

Building on this work, the present study investigates the individual variability of functional language networks in patients with brain tumors located in or near language-related areas by comparing patients’ fingerprints to a normative healthy reference fingerprint. Extending previous task-based implementations, functional fingerprints will be derived from resting-state functional magnetic resonance imaging (fMRI) data, which offers a key advantage in tumor populations where functional impairments frequently limit task compliance (Kumar et al., 2020). Patients are expected to show highly individualized functional reorganization trajectories, ranging from alignment with the normative network structure to diverse patterns of deviation. Changes in connectivity fingerprints are tracked relative to language performance from pre-to postoperative stages and at three-month follow-up. It is hypothesized that aberrant fingerprint patterns are associated with language deficits. An assimilation toward the normative network may reflect successful treatment effects or adaptive reorganization processes. If successful, this approach could offer an innovative tool to advance the understanding and monitoring of language network reorganization in patients with brain tumors.

## 2 Methods

### 2.1 Study procedure and participants

A total of 27 patients with a left-hemispheric brain tumor located in or near language-related areas (see Supplementary Figure S1 for a heatmap of tumor location) were included in this study. Patients undergoing elective tumor resection between August 2023 and December 2024 were prospectively screened for eligibility. Inclusion criteria were: (1) right-handedness, (2) left-hemispheric tumor in or near language-relevant areas, and (3) German as primary language. Patients under 18 years of age or with cognitive impairment precluding informed consent were excluded. No post-hoc exclusions based on outcome data were performed. Data were collected at up to three time points: preoperatively, in the immediate postoperative phase and at a three-month follow-up. Preoperative scans corresponded to the data used for intraoperative planning (*M* = 21.9 days, *SD* = 22.73, range = 1 – 86); postoperative and follow-up scans were obtained at a mean of 1.8 days (*SD* = 1.42, range = 1 – 3) and 103 days (*SD* = 11.6, range = 83 – 122) after surgery, respectively. Postoperative imaging data were available for 25 patients, while twelve patients completed the three-month follow-up scan. Missing immediate postoperative imaging data resulted from patients being scanned on a different scanner within our institution due to clinical workflow constraints. Missing three-month follow-up data resulted from patients undergoing routine follow-up imaging at external institutions. No loss to follow-up was attributable to death or disease progression. Refer to Table 1 for descriptive data of patients. To establish a reference for a healthy network structure, 30 right-handed, German-speaking healthy participants (15 women, 15 men; age: *M = *36.63 years, *SD* = 17.46) underwent two scanning sessions three months apart (*M = *91.97 days, *SD* = 14.1). All participants had no history of neurological or psychiatric disorders. Healthy controls were recruited through local advertisements and personal referrals and on average younger than patients (*p* < .001).

**Table 1:** Patient characteristics.

Patient characteristic	Number of patients
N	27
Sex (female/male)	7/20
Mean age at preoperative time point in years (min; max)	52.15 (31; 73)
Awake surgery	20
Previously resected recurrence	7
Preoperative tumor volume in cm^3^ (min; max)	36.6 (3.0 – 100.2)
*Tumor location*	
Frontal lobe	8
Temporal lobe	10
Parietal lobe	5
Insula-involving	4
*Pathology*	
Oligodendroglioma (WHO 2, 3)	4
Astrocytoma (WHO 2, 3, 4)^a^	6
Glioblastoma (WHO 4)	13
Dysembryoplastic Neuroeptihelial Tumor (WHO 1)	1
Metastasis	3

Note.

a for one patient, WHO grade (2 vs. 3) could not be definitively assigned due to borderline histological features.

For exploratory subgroup analyses, patients were categorized into low-grade glioma (LGG) and high-grade glioma (HGG) groups based on a combination of histopathological and molecular criteria (Eichinger et al., 2017; Louis et al., 2016; Wu et al., 2020). IDH-mutant diffuse gliomas (including astrocytomas and oligodendrogliomas, WHO grades 2–4) were grouped together (n = 10), and IDH-wildtype glioblastomas (WHO grade 4) formed the second group (n = 13). No IDH-wildtype grade 3 gliomas are present in the sample. Adjuvant chemoradiotherapy was initiated 1–2 months after surgery in all patients except those with LGG. Patients with LGG (*M* = 45.55 years, *SD* = 10.45) were significantly younger than those with HGG (*M* = 55.31 years, *SD* = 8.16; *U* = 116.00, *p* = .009) but did not significantly differ in tumor volume (LGG: *M* = 46.26 cm^3^, *SD* = 33.84; HGG: *M* = 35.68 cm^3^, *SD* = 28.70; *U* = 55.50, *p* = .361.) Four LGG and three HGG cases were recurrences.

### 2.2 Regions of interest

**Fig. 1:**
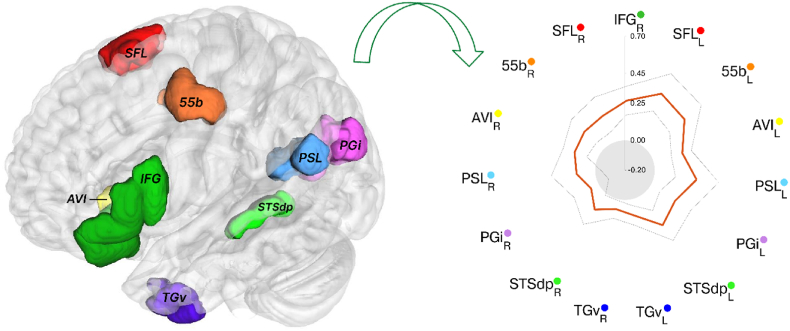
Left figure displays the selected Regions of Interest (ROIs) shown in the left hemisphere. Region color coding: green = IFG (inferior frontal gyrus, areas 44, 45, 47l); orange = 55b (premotor area); red = SFL (Superior Frontal Language Area); light green = STSdp (dorsal posterior superior temporal sulcus); blue = PSL (Perisylvian Language Area); pink = PGi (inferior parietal gyrus); dark purple = TGv (ventral temporal gyrus); yellow = AVI (anterior ventral insula). Right figure displays the healthy fingerprint template. The axis represents z-normalized correlation values from the seed to individual targets. The subscript L (left hemisphere) or R (right hemisphere) denotes the hemisphere in which the target is located. Orange line: mean control fingerprint values at first scanning session; grey lines: standard deviation.

Regions of interest (ROI) were defined using the HCP-MMP1.0 atlas by Glasser et al. (2016). The inferior frontal gyrus (IFG) was selected as seed region due to its key role in both language production and comprehension (Ishkhanyan et al., 2020; Price, 2012). To form the IFG, the three parcellated regions of the HCP-MMP1.0 atlas pars opercularis (44), pars orbitalis (47l) and pars triangularis (45) were added. The seed was placed in the left (ipsilesional) hemisphere. The selection of target ROIs was guided by complementary criteria. Regions were primarily selected based on their reported effective connectivity with IFG-related language regions in Rolls et al. (2022), who systematically characterized parcel-level connectivity within the Glasser atlas (Glasser et al., 2016). Targets were distributed across frontal, temporal, and parietal cortex to obtain a spatially broad fingerprint and selected to ensure topographic variability in the resulting fingerprint profile (see Supplementary Table S1 for details). To assess both intra- and interhemispheric connectivity of the seed region, seven left-hemispheric cortex areas and their right hemisphere homologues were chosen as targets. These regions are for each left and right hemisphere: Superior Frontal Language Area (SFL), anterior ventral insula (AVI), ventral temporal gyrus (TGv), inferior parietal gyrus (PGi), dorsal posterior superior temporal sulcus (STSdp), Perisylvian Language Area (PSL), and the premotor area 55b. Additionally, the right-hemisphere homologue of the IFG (parcels 44, 45, and 47l combined) was included as a target region. ROIs are shown in Fig. 1. Details of their selection are provided in Supplementary Table S1. For ROI generation in native space, data were preprocessed with FreeSurfer (Fischl, 2012); for patients, T1-weighted images with manually drawn tumor masks for lesion filling were used as input. The HCP-MMP1.0 atlas was registered to each subject's anatomical space and converted into a volumetric parcellation. All ROI registrations were visually inspected to verify adequate alignment of cortical landmarks and parcellation boundaries, particularly in regions adjacent to the tumor. For subsequent analyses, ROIs were slightly dilated in anatomical space before registration to each individual's functional space to ensure complete representation after resampling.

### 2.3 Magnetic resonance imaging (MRI)

All imaging sessions for both patients and healthy controls were conducted on the same scanner (Siemens 3T Prisma, 64 channel head coil) using identical acquisition protocols. For all participants, high-resolution T1-weighted structural images (1 mm isotropic voxels), fluid-attenuated inversion recovery (FLAIR) images, and resting-state functional magnetic resonance imaging (fMRI) data based on blood oxygenation level–dependent (BOLD) contrast (gradient-echo EPI sequence) were acquired. Further details are provided in the Supplementary Methods. In patients, the sequences were obtained as part of the clinical protocol.

### 2.4 Functional preprocessing

Imaging data underwent visual quality control before preprocessing. Raw functional data were evaluated for signal dropout, ghosting, and susceptibility-related distortions, with signal quality assessed specifically within the seed and target ROIs used for connectivity analysis. Scans were flagged for exclusion if the seed region was rendered unusable due to extensive signal dropout or susceptibility artifacts beyond what could be addressed by voxel-wise exclusion; however, no scans met this criterion. Structural scans were additionally reviewed, with particular attention given to the immediate postoperative scans regarding pneumocephalus and perioperative edema; lesion masks were drawn accordingly to account for affected tissue. Co-registration was verified using anatomical landmarks. Resting-state fMRI data were preprocessed in line with Pruim et al., 2015a, Pruim et al., 2015b using FMRIB Software Library's (FSL; Jenkinson et al., 2012) tools as follows: Removal of the first five volumes for signal equilibration, volume realignment to the middle volume using MCFLIRT to correct for head movement, spatial smoothing with a 6 mm FWHM kernel, affine boundary-based registration of resting-state data to each participant's structural image using FLIRT. Next, ICA-AROMA was applied in non-aggressive mode to remove motion artifacts. Subsequently, mean white-matter and cerebrospinal fluid time series were regressed out to further reduce residual structured noise (Ciric et al., 2017; Pruim et al., 2015). Global signal regression was not applied, as it can introduce artifactual anticorrelations in seed-based connectivity analyses and may distort comparisons between groups or conditions when the global signal differs systematically (Murphy et al., 2009; Gotts et al., 2020). Finally, a temporal high-pass filter (cutoff = 0.01 Hz) was applied to remove low-frequency drifts. As the fingerprinting analysis assesses the connectivity between a seed area and chosen target regions a functional connectivity map was generated using seed-based correlation analysis (SCA) as described (Voets et al., 2019, 2021): The mean fMRI timeseries was extracted from the seed mask and correlated with every voxel in the brain, yielding whole-brain correlation maps which were standardized using Fisher z-transforms (see Supplementary Figure S2). Subsequently, the target regions were overlaid onto the whole-brain correlation maps, and mean z-normalized correlation values were extracted for each target region. These values are further used for both statistical analyses and the visualization of a fingerprint. For each ROI, voxels overlapping with tumor or edema tissue were removed from the analysis to avoid the inclusion of erroneous timeseries. The minimum number of remaining voxels per region was determined using sample size calculation with Finite Population Correction (Israel, 1992) to ensure that, despite the exclusion of voxels a valid statement about the entire region can still be made with 95% statistical confidence. ROIs with insufficient remaining voxels were excluded from the analysis. As tumor and edema masks were registered across all measurement time points and combined, longitudinal comparisons were always based on the same set and number of targets (see Supplementary Methods for more information).

### 2.5 Statistical fingerprint analysis

The statistical comparison of fingerprints was carried out using MATLAB scripts from the MR Comparative Anatomy Toolbox (MrCat) which are freely available for download (www.neuroecologylab.org). Following Mars et al. (2016), Manhattan Distance (MD) was used as distance metric to compare fingerprints following the formula $D=\sum_{i=1}^{n}\left|p_{i}-q_{i}\right|$, where *p* and *q* represent the two fingerprints and *i* indexes the *n* target regions. Smaller distance values indicate a higher degree of similarity between the fingerprints. Prior to analysis, for each fingerprint, connectivity values were normalized to a range from 0 to 1, where 0 represents the weakest and 1 the strongest seed-to-target connection, resulting in a contrast of connectivity patterns rather than absolute values (2016). Patients' fingerprints are compared both to a healthy network template and longitudinally within individuals across the three measurement time points. Primary inference targets preserved or restored alignment of a patient's network with the normative language architecture. Following Voets et al. (2019), deviation and reorganization is not presumed to occur by default in all patients. Accordingly, the a priori expectation is that (i) an individual patient's fingerprint matches the normative language-network template, and (ii) within-subject fingerprints match across sessions unless change in network organization has occurred. Such group-averaged healthy network references have been successfully applied across neurological and psychiatric disorders to identify clinically relevant connectivity abnormalities (Sha et al., 2018). Permutation testing (10,000 permutations) is used to assess whether the MD is significantly smaller than expected by chance, thereby determining whether (i) a patient's language network matches the normative structure (interpreted as typical) or no longer aligns with it (interpreted as atypical) and (ii) whether it remains stable over time (‘match’) or shows change (‘non-match’). For a more detailed description of the statistical procedure, see Mars et al. (2016). For subsequent statistical analyses, MD values were normalized by dividing each value by the number of target regions, ensuring comparability across patients despite exclusion of targets affected by tumor or edema. All values were subsequently z-standardized to the healthy control group, with normative mean and standard deviation calculated from the same subset of target regions as available in each patient. To quantify hemispheric asymmetry of a connectivity fingerprint, a lateralization index (LI) was calculated as $\text{LI}=\frac{L-R}{\left|L\right|+\left|R\right|}\text{,}$ where L and R denote the mean connectivity strengths with targets in the left and right hemisphere (Seghier, 2008). The LI reflects the relative lateralization of seed connectivity, with positive values indicating a leftward and negative values a rightward bias. All analyses were conducted using predefined processing and statistical pipelines applied uniformly across all subjects and time points. No repeated experimental runs or technical replicates were performed.

### 2.6 Neuropsychological assessment

Patients underwent a comprehensive neuropsychological assessment prior to surgery, including linguistic and non-linguistic tests. Neuropsychological assessments were administered by trained neuropsychologists following standardized test instructions. The present study focused on the results of the language assessments Aachener Aphasia Test (AAT; Huber et al., 1983) and Regensburger Word Fluency Test (RWT; Aschenbrenner et al., 2000), while the Trail Making Test (TMT; Reitan, 1958) was included as a sensitivity measure. All test scores were calculated according to the scoring rules and normative data specified in the respective test manuals. AAT subtests were grouped into language production, comprehension, and written language, with deficits defined as scores below a percentile rank (PR) of 89.4. For verbal fluency, phonological and semantic subtests were combined into separate fluency scales (Cronbach's α > .90); a PR ≤ 10 was classified as a deficit. For statistical analyses, RWT and TMT scores were z-standardized relative to the control distribution. At both postoperative time points and during the two control group sessions, a shortened version of the preoperative neuropsychological assessment was conducted which included the AAT, RWT, and TMT. Neuropsychological assessments and imaging data analyses were conducted independently and at separate time points.

### 2.7 Statistical analysis

Statistical analyses were conducted using SPSS (v29) and MATLAB (R2015b). A linear mixed-effects model (LMM) served as the primary analytical strategy for repeated-measures data, with subject included as a random factor and a Toeplitz covariance structure. LMMs were applied to MD and language performance to assess group differences, longitudinal changes within patients, and the impact of clinical variables. Where LMMs failed to converge, linear regressions were run separately for each time point. Model assumptions were checked using Q-Q plots and residual plots. To account for small or unequal group sizes and ordinal data, nonparametric tests were employed: Wilcoxon rank tests were applied for within-patient comparisons of AAT, Mann–Whitney U tests for group comparisons between LGG and HGG and Kruskal–Wallis tests for comparisons across tumor locations. Analyses involving the lateralization index were conducted using linear regression and Pearson correlations. The significance threshold was set at p < .05. Benjamini–Hochberg FDR correction was applied to control for multiple comparisons (Benjamini and Hochberg, 1995). The statistical analysis plan was defined prior to data analysis; no exploratory analyses were reclassified as confirmatory.

### 2.8 Ethics

The study was approved by the Ethics Committee of the Medical University of Graz (EK-Nr. 27-287 ex 14/15) and conducted in accordance with the principles of the Declaration of Helsinki. All participants gave informed written consent to take part.

## 3 Results

### 3.1 Temporal stability of fingerprints and healthy template generation

Session effects were assessed using a paired permutation test (10,000 permutations) on the distance between mean control fingerprints at session 1 and session 2. No significant difference was observed, indicating no systematic drift across sessions (*p* = .99). In addition, temporal stability at the individual level was assessed in healthy individuals by quantifying the MD between fingerprints obtained three months apart using a permutation test. Fingerprints proved to be temporally stable. Of 29 participants, 28 (96.6%) exhibited a significant intra-subject match even after alpha error correction (*p* = .01–.045; one non-significant case: *p* = .12). One participant was excluded from this analysis due to unintended music playback during the second session, a factor that may affect resting-state connectivity (Kay et al., 2012). To create a normative healthy template, fingerprints of controls from the first scanning session were averaged. The resulting template is illustrated in Fig. 1. As a sanity check, fingerprints from the second session of controls were tested against this template. All controls showed a significant match with the normative template (all p-values <.03).

### 3.2 Language performance

Mixed-model analyses revealed significant group × time interactions for both semantic (*F*(2, 40.54) = 5.16, *p = *.028, *η*^*2*^_*p*_ = .20; age-controlled) and phonological fluency (*F*(2, 32.94) = 18.35, *p* < .001, *η*^*2*^_*p*_ *= *.53; age-controlled). Across time, patients performed significantly worse than controls (semantic: *F*(1, 26.48) = 7.11, *p* = .013, *η*^*2*^_*p*_ *= *.21; phonological: *F*(1, 26.86) = 16.11, *p* < .001, *η*^*2*^_*p*_ *= *.38). Controls showed stable semantic fluency between baseline and follow-up, whereas phonological performance improved significantly over time (*p* < .001), attributable to practice-related increases. Within the patient group, postoperative declines were evident in both fluency tasks (semantic: *F*(2, 25.43) = 16.21, *p* < .001, *η*^*2*^_*p*_ *= *.56; phonological: *F*(2, 21.82) = 7.25, *p* = .004, *η*^*2*^_*p*_ *= *.40). Semantic fluency remained impaired at follow-up, whereas phonological fluency showed partial recovery by three months, with no significant difference relative to either pre- or postoperative performance. Postoperative declines in patients were corroborated by AAT findings, with significant decreases in language production (*Z* = −2.92, *p* = .003, *r* = −.57), comprehension (*Z* = −3.25, *p* = .001, *r* = −.64), and written language (*Z = *−2.82, *p* = .005, *r* = −.55). No significant changes were observed between postoperative or preoperative and follow-up assessments (all p-values >.12). Descriptive statistics are reported in Supplementary Table S2.

In group comparisons of HGG versus LGG, no significant preoperative differences were found in AAT or RWT subtests (all p-values >.26). Immediately after surgery, HGG patients performed significantly worse than LGG patients in phonological fluency (*U* = 17.0, *p* = .007, *r* = −.59), written language (*U* = 20.5, *p* = .014, *r* = −.54) and language production (*U* = 17.5, *p* = .007, *r* = −.59), while no group difference was observed for language comprehension (*U = *35.0, *p* = .154, *r* = −.31) and semantic fluency (*U* = 31.0, *p* = .090, *r* = −.37). Comparisons between patients with recurrent and newly diagnosed tumors revealed no significant differences in language performance pre- or postoperatively (all p-values >.34). Group comparisons were not conducted at follow-up due to the small number of patients with LGG or recurrent tumors available.

### 3.3 Alterations in connectivity patterns

**Fig. 2:**
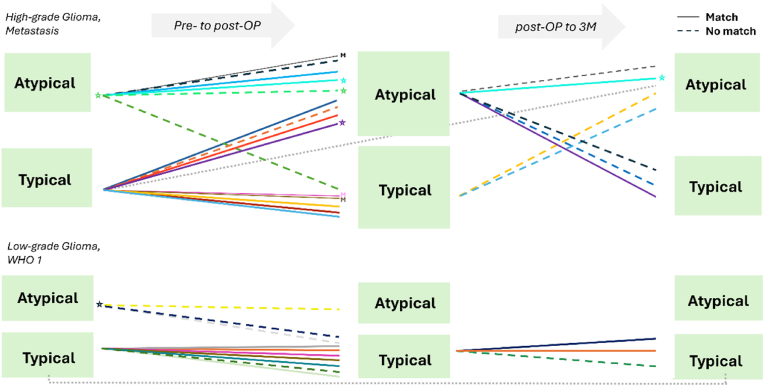
Fig. 2 illustrates changes in the binary fingerprint classification across the three time points as well as intra-subject comparisons between time points. Each color represents a single patient; solid lines indicate an intra-subject match between two patient's fingerprints, dashed lines indicate a non-match. Stars indicate, that a patient's fingerprint became atypical after correction for multiple comparisons. High-grade glioma and metastases are shown in the upper panel, low-grade glioma in the lower panel. Metastases are labeled with an "M" and shown in black, light pink and brown.

**Fig. 3:**
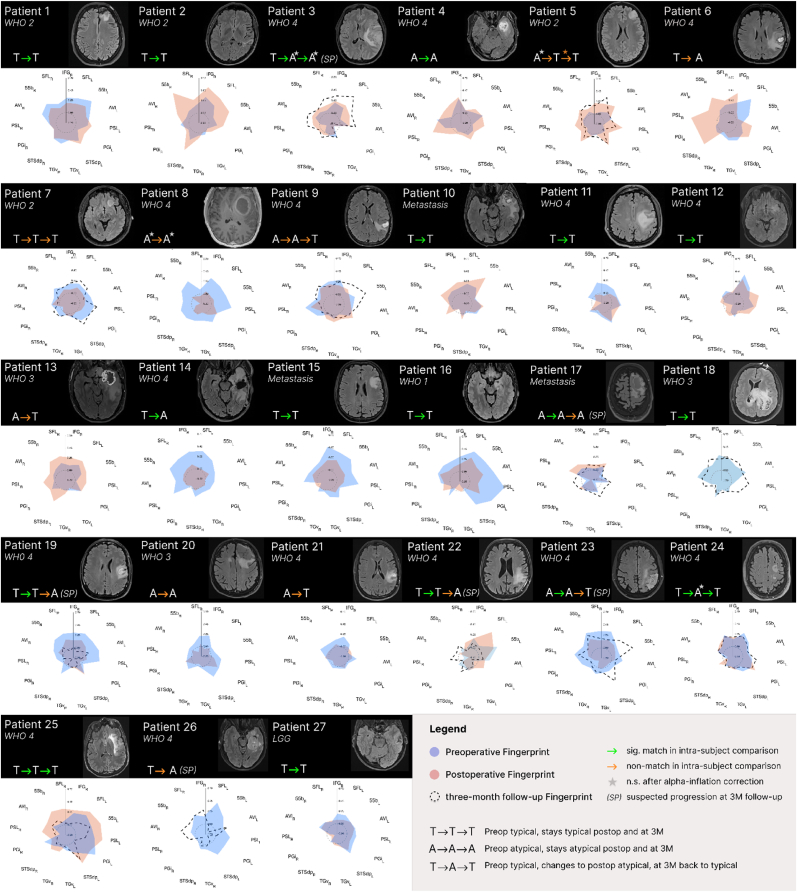
Overview of patient fingerprints and their changes across time points.

Patients did not differ significantly across the three time points in their deviation from the healthy template (*F*(2, 31.535) = 0.583, *p* = .564, *η*^*2*^_*p*_ *= *.04; age-controlled; similarly non-significant when restricted to primary CNS tumors, *p* = .641; see Supplementary Table S3). Descriptively, deviations increased postoperatively and remained elevated at follow-up (see Supplementary Table S2). Preoperatively, fingerprints did not match the typical network in 9/27 patients (33%, 3 ‘low grade’ and 5 ‘high grade’, 1 ‘metastasis’, 2 previously resected). Directly after surgery, 10/25 patients (40%, 1 ‘low grade’ and 8 ‘high grade’, 1 ‘metastasis’, 2 previously resected), and three months after surgery, 5/12 patients (42%, 0 ‘low grade’ and 4 ‘high grade’, 1 ‘metastasis’, 1 previously resected), did not match the typical network. Changes in the binary fingerprint classification (typical/atypical) were highly individual, both from pre-to postoperative assessments and from postoperative to the three-month follow-up. Some patients showed stable fingerprints across all time points, whereas others exhibited marked shifts toward more typical or more atypical network configurations after surgery or during follow-up. High individuality was likewise seen in the longitudinal intra-subject comparisons. Changes in fingerprint classification over time are shown in Fig. 2, while Fig. 3 provides an overview of all patients and their fingerprint profiles. MD values across time points are listed in Supplementary Table S4 and visualized in Figure S3.

### 3.4 Data quality assessment and control analyses of fingerprint robustness

To ensure comparable data quality across measurement time points, framewise displacement (FD) and temporal signal-to-noise ratio (tSNR) were assessed. Mean FD did not differ between preoperative, postoperative, and follow-up scans (*F*(2, 27.90) = 0.36, *p* = .702). Moreover, mean tSNR across ROIs did not differ between time points, both for all ROIs (*F*(2, 32.71) = 0.61, *p* = .548) and for ipsilesional targets only (*F*(2, 33.61) = 1.20, *p* = .313). Within the seed region specifically, tSNR likewise did not differ across time points (*F*(2, 29.61) = 0.341, *p* = .713). To further rule out that signal quality drove fingerprint deviations, additional analyses examined associations between seed-region tSNR, mean ROI tSNR, FD, and both MD and binary fingerprint classification. None of these analyses revealed significant associations (see Supplementary Tables S5 and S6). Since voxels overlapping with tumor or edema tissue were excluded from the ROI, it was examined whether a reduction in seed size would lead to a greater deviation from the healthy template and thereby increase the probability of an atypical fingerprint. To this end, we re-analyzed all 36 patient - time point observations with typical fingerprints by artificially reducing seed size through erosion until the resulting seeds fell below the average seed size observed in atypical cases with tumor or edema overlap. MD to the healthy template was recalculated, and fingerprints were reclassified as typical versus atypical. All fingerprints remained typical after seed size reduction (p-values <.045). Additionally, to test whether reducing seed size altered the resulting distance values beyond the binary typical/atypical classification, we compared MD values from the reduced seeds with those obtained in the main analysis using an equivalence test (two one-sided tests, TOST). MD values derived from the smaller seed were statistically equivalent to those from the main analysis (equivalence margin ±2.5% of the original mean). Both one-sided tests were significant (*p* = 0.017 and *p* = 0.026), indicating that the difference fell within the predefined equivalence bounds. Thus, seed size did not meaningfully affect the fingerprint structure, and atypicality was not attributable to seed size reduction. Supplementary Figure S4 illustrates example fingerprints before and after reduction. Finally, analyses revealed no significant association between the number of excluded targets and atypical fingerprint classification (Fisher–Freeman–Halton exact tests: preoperative *p* = .377; postoperative *p* = .120). Likewise, the number of excluded targets was not significantly associated with MD (preoperative: *r*_*s*_ = .338, *p* = .085; postoperative: *r*_*s*_ = .159, *p* = .447). No consistent pattern regarding the identity of excluded targets was observed between typical and atypical fingerprints (see Supplementary Table S7).

### 3.5 Language performance and fingerprints

**Fig. 4:**
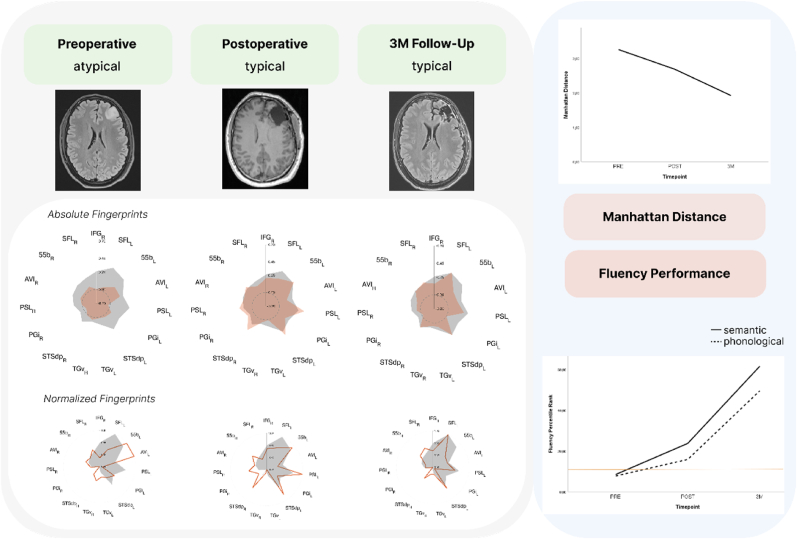
Example of a patient with a left frontal oligodendroglioma (WHO grade 2) and no signs of tumor progression at the three-month follow-up. Left panel: top row shows structural MRI scans at the three time points; subsequent rows display absolute (top) and normalized (bottom) fingerprints, with each axis representing the (relative) connectivity of the seed to a target region. Right panels: longitudinal changes in Manhattan Distance (top) and semantic and phonological fluency performance (bottom). The orange line in the fluency panel denotes the clinical cut-off for impairment. Preoperatively, the patient exhibited an atypical fingerprint (*p* = .15) and deficits in word fluency. Postoperatively, the fingerprint shifted toward a typical pattern (*p* = .01), with further assimilation at the three-month follow-up (*p < *.01), as indicated by the reduction in Manhattan Distance (all results alpha-error corrected). These changes paralleled improvements in fluency performance across time points. Reductions in Manhattan Distance thus mirrored the clinical course, tentatively suggesting an adaptive assimilation process in this patient. Intra-subject comparisons revealed no match between pre- and postoperative (*p* = .42) or pre- and three-month assessments (*p* = .37), indicating that change occurred. The comparison from postoperative to follow-up was marginal, showing a match before but not after alpha-error correction (*p* = .09).

Patients with deficits in language production, comprehension, and/or semantic fluency deviated more strongly from the typical network structure than patients without such deficits (all *p* < .05, age-controlled). No significant group differences were found for written language or phonological fluency (see Table 2 for results). At the immediate postoperative time point, higher MD significantly predicted worse phonological fluency (β = −.67, *p* = .002; age-controlled; see Supplementary Figure S5). The model predicting semantic fluency did not reach significance; however, a trend was observed, and MD emerged as a significant negative predictor within the model (β = −.48, *p* = .040; age-controlled). Preoperatively and at three-month follow-up, regression coefficients also indicated negative associations between network deviation and both fluency measures, but none of these reached significance. Full statistical results are provided in Table 3. Sensitivity analyses restricted to primary CNS tumors yielded comparable results and are reported in Supplementary Table S3 and S8. To explore the relationship between binary fingerprint classification and language deficits, we descriptively examined the presence of deficits across typical and atypical profiles. Deficits were more frequent among atypical than typical profiles (AAT: 64% vs. 30%; semantic fluency: 64% vs. 35%), whereas phonological fluency showed similar rates in both groups (60% vs. 56%). An illustrative case example is presented in Fig. 4. To assess domain specificity, we examined whether MD was associated with non-linguistic cognitive measures. MD was not significantly associated with TMT-A or TMT-B performance at either the pre- or postoperative time point (all *p* ≥ .461, all |β| ≤ .11).

**Table 2:** Statistical results of Linear Mixed Models (LMM).

Test	*F*(df)	*p*	*M* (Deficit) [95% CI]	*M* (noDeficit) [95% CI]	*η*^*2*^_*p*_
Language production	*F*(1,24.53) = 5.69	.025∗	2.35 [1.51, 3.19]	1.11 [0.55, 1.67]	0.188
Language comprehension	*F*(1,15.36) = 11.24	.018∗	3.17 [1.90, 4.44]	1.01 [0.25, 1.78]	0.423
Written language	*F*(1,32.77) = 0.39	.538	1.39 [0.78, 2.79]	1.78 [0.71, 2.07]	0.012
Semantic fluency	*F*(1,15.23) = 6.97	.018∗	2.28 [1.56, 3.01]	0.73 [-0.07, 1.54]	0.314
Phonological fluency	*F*(1,30.27) = 0.06	.807	1.68 [0.75, 2.30]	1.52 [0.75, 2.60]	0.002

*Note.* All results are age-controlled. Linear Mixed Models (LMM) were conducted with Manhattan Distance (MD) as dependent variable; means represent estimated marginal means. Partial eta squared (η^2^_p_) was calculated from the F statistics using η^2^_p_ = F/(F + df_error). ∗*p* < .05, ∗∗*p* < .01.

**Table 3:** Statistical results of regression analyses*.*

Time point	Fluency Subtest	*F*(df)	*p*	*R*^*2*^ (adj.)	β	*t*	*p* (β)
Pre	Phonological	*F*(2,21) = 2.05	.154	.163 (.084)	−.37	−1.81	.184
	Semantic	*F*(2,21) = 1.03	.375	.089 (.002)	−.26	−1.20	.243
Post	Phonological	*F*(2,21) = 7.86	.003∗∗	.428 (.374)	−.67	−3.56	.002∗∗
	Semantic	*F*(2,21) = 2.76	.086	.208 (.133)	−.48	−2.19	.040∗
3M FU	Phonological	*F*(2,9) = 1.54	.272	.278 (.097)	−.09	−0.25	.807
	Semantic	*F*(2,9) = 0.60	.573	.130 (−.088)	−.08	−0.21	.840

*Note.* All results are age-controlled. Regression analyses were conducted with Manhattan Distance (MD) as predictor. ∗*p* < .05, ∗∗*p* < .01. Pre = preoperative time point; Post = postoperative time point; 3M FU = three-months follow-up.

To further explore connectivity fingerprints in relation to language, changes in the laterality index (LI) from pre-to postoperative time points were examined on an exploratory basis. In HGG patients, a greater rightward shift in LI significantly predicted smaller declines in phonological fluency after surgery (β = −0.82, *t* = −4.6, *p* = .002; not significant in the full sample: β = −0.43, *p* = .083; both age-corrected). Sensitivity analyses of connectivity for individual regions revealed no significant effects, though trend-level correlations emerged across three right-hemispheric regions (AVI_R_: *r* = .57, *p* = .056; PGi_R_: *r* = .55, *p* = .064; STSdp_R_: *r* = .53, *p* = .073). No significant associations were found between LI change and semantic fluency (HGG: β = −0.55, *p = *.100; full sample: β = −0.19, *p* = .46; both age-corrected). Exploratory descriptive inspection of LI at the three-month follow-up revealed apparent trends related to tumor progression status. All six patients without tumor progression showed left-lateralized LI (.35-.69), each exhibiting further leftward shifts and stable or improved language performance relative to the preceding assessment. In contrast, five of six patients with suspected progression exhibited right-lateralized connectivity at follow-up (LI = – .14 to −.01; one left-lateralized, LI = .29), with a language decline in three patients.

### 3.6 Influence of tumor grade, age, tumor location, tumor volume, tumor–seed overlap and tumor recurrence on fingerprints

Regarding tumor grade, patients with LGG exhibited more typical fingerprints (19/23) than HGG, with one of the four atypical cases identified only after correction for multiple comparisons. In contrast, HGG patients exhibited a more heterogeneous pattern (15 typical, 18 atypical; four atypical cases identified only after correction). Changes in binary classification, whether through assimilation or deviation from the typical network, were predominantly observed in HGG patients (see Fig. 2). Postoperatively, HGG patients demonstrated significantly greater network deviation than LGG patients (*U* = 93.00, *p* = .030, *r* = .46), whereas preoperatively this difference did not reach significance (*U* = 80.00, *p* = .622, *r* = .10). Groups were not compared at three months due to small subgroup sizes. Regarding seed–tumor/edema overlap: overlap was present in 9/27 patients preoperatively, 12/25 postoperatively, and 6/12 at three-month follow-up. The rate of atypical fingerprints was comparable between cases with and without overlap (without overlap: 13/33 (39%) fingerprints atypical; with overlap: 11/31 (35%) fingerprints atypical). Neither the binary presence of overlap nor the percentage of seed–tumor overlap were significantly associated with MD values at any time point (see Supplementary Table S9 for full results). Seed tSNR did not differ significantly between patients with and without overlap (*F*(1, 46.78) = 0.55, *p* = .461). Age did not show a significant effect on MD, *F*(1, 21.04) = 1.15, *p = *.29, *η*^*2*^_*p*_ *= *.05. Further analyses revealed no significant effects of tumor location, tumor volume, or recurrence status on MD (all p-values >.05; see Supplementary Table S10 for details).

## 4 Discussion

Language processing in the human brain relies on a widely distributed network that is vulnerable to disruption (Fedorenko et al., 2024; Krishna et al., 2023; Moretto et al., 2023). The present study set out to examine how brain tumors and subsequent treatment affect the organization of the language network, characterizing both deviations from the typical structure and intraindividual trajectories of reorganization. The results demonstrate unique connectivity patterns across patients, that changed in various ways: some patients showed an assimilation to the healthy network structure over time, whereas others exhibited more pronounced deviations, differing in both their nature and extent. This individual variability could be revealed by applying a single-subject fingerprinting approach (Mars et al., 2016), which overcomes the limitations of group-level analyses that mask individual differences — differences that are, in fact, to be expected in tumor populations given the heterogeneity in clinical variables such as tumor location, progression, and treatment.

Beyond characterizing network organization, the present study demonstrates that functional connectivity fingerprints provide a means of gaining insight into patients' language performance. Greater deviations from the typical network structure were systematically associated with the presence of deficits across core language domains, including language production, comprehension, and semantic fluency. This finding underscores that, consistent with previous work, not only absolute connectivity strength, but also the overall network architecture, carries information relevant to language function (Sun et al., 2024; Yuan et al., 2020). While it was initially hypothesized that greater network deviation from the normative healthy structure would be consistently linked to poorer language performance across time points, the results indicated time-varying associations: Greater deviation from the typical network predicted poorer phonological fluency only immediately after surgery, with a similar trend for semantic fluency. No such association was observed preoperatively or at the three-month follow-up, though the latter should be interpreted cautiously given the reduced sample size at that time point. Compared to the preoperative assessment, the immediate postoperative phase was marked by a significant decline in language performance and descriptively higher network deviations. Although the latter increase did not reach significance, the pattern aligns with prior reports of transient disruptions in functional network properties during the early postoperative period, mostly attributed to surgery- or treatment-related influences such as anesthesia, edema, or fatigue (Bharath et al., 2017; Chee and Zhou, 2019; Stoecklein et al., 2023). A temporary decline in language function has likewise been reported during this phase (Dallabona et al., 2017; Zigiotto et al., 2020, 2022). For instance, Yuan et al. (2022) observed reduced functional integration within the language network of glioma patients two weeks after surgery, accompanied by a transient decline in language performance*,* with both measures showing recovery by the three-month follow-up. In our cohort, recovery was partially evident at three-month follow-up, with language performance tending to return toward preoperative levels, whereas no corresponding effect was observed in Manhattan Distance (MD). Patient-specific variability in the disease course likely played a major role at this stage, presumably masking effects at the group level; however, given the reduced sample completing the follow-up assessment, these observations remain exploratory. Nevertheless, the findings do reinforce the notion that the immediate postoperative phase constitutes a period of heightened vulnerability for language processes and, at least descriptively, their supporting networks. Importantly, associations with non-linguistic variables did not reach significance, suggesting that the observed relationship is not simply indicative of a general cognitive decline but more specific to language performance. The enhanced association immediately after surgery may therefore either reflect short-term surgery- or treatment-related factors or indicate time-dependent (mal-)adaptive network dynamics, implying a direct relationship between network deviation and language performance. While causal inference is constrained in most studies due to surgery-related confounds, the (mal)adaptive nature of a network structure warrants emphasis because of its methodological implications for fingerprinting. Deviations from the healthy network structure should not be interpreted as inherently adverse, as they may also reflect compensatory mechanisms supporting language function (Hartwigsen et al., 2017; Kiran and Thompson, 2019; Pasquini et al., 2022). If immediate postoperatively reorganization is inefficient, i.e. maladaptive, deviations from the typical pattern are more likely to be associated with poorer language performance. By contrast, if deviations of similar magnitude observed preoperatively or at follow-up reflect less maladaptive or even adaptive reorganization, their association with language performance can be expected to be weaker or even reversed. Crucially, MD captures only the extent of deviation rather than the specific manner in which a network deviates, and thus provides no information about whether changes are adaptive or maladaptive. For example, in one patient, MD remained nearly identical before and after surgery, yet the underlying network pattern changed substantially. Notably, in this case a language deficit emerged only postoperatively (see Supplementary Figure S6). This suggests that sole reliance on global distance metrics may not be sufficient to reliably predict performance, as it risks overlooking clinically relevant aspects of a pattern's (mal)adaptivity. This likewise applies to the binary classification of deviation, underscoring that fingerprints should be interpreted not only as a “deficit detector” based on a typical-atypical distinction, but rather complemented by analysis of the entire network pattern. Accordingly, a key next step for future research will be to establish markers of (mal)adaptive fingerprint patterns to enable more nuanced clinically interpretations.

Two further methodological considerations can be derived from the case example presented in Fig. 4. First, the case highlights aspects regarding the fingerprint's sensitivity: Despite deficits in word fluency, the patient's preoperative fingerprint was only classified as atypical after correction for multiple comparisons, placing it at the borderline between typical and atypical. Although intra-subject comparisons did reveal a change in the pattern from pre-to postoperative, this suggests a reduced sensitivity of the fingerprint to capture clinically relevant alterations. Similar cases were observed in our sample with fingerprints being classified as typical despite the presence of language deficits. Atypical fingerprints in patients without deficits, by contrast, were rare (four cases across all time points). Importantly, these are also not necessarily misclassifications, as atypical patterns may arise from adaptive reorganization supporting language performance. The sensitivity–specificity balance of a fingerprint is inherently dependent on the ROI selection. Because the fingerprint is normalized to a 0–1 range, the distribution of raw connectivity values across targets shapes the topography of the profile and thereby determines its discriminative capacity. If all selected targets exhibit similarly strong connectivity with the seed, the raw values cluster within a narrow range, and after normalization, minimal differences are stretched across the full 0–1 scale. A key factor in the sensitivity–specificity balance of the present fingerprint pattern is the inherent hemispheric asymmetry, with stronger left- and weaker right-hemispheric connectivity to the seed, best visible in the normalized template. This hemispheric asymmetry represents a specific instance of the general principle described above: with bilateral targets and a left-hemispheric seed, in the most extreme case, if all targets within each hemisphere exhibit near-identical values, the normalized profile reduces to a binary hemispheric contrast; a pattern that is largely shared across all individuals and carries minimal discriminative information. Although this pattern unlikely occurs in such an extreme form, the left–right contrast in the present data nevertheless tends to increase statistical significance, as alignment with the template is driven to a considerable extent by global hemispheric asymmetry, which in turn reduces the fingerprint's sensitivity to subtle but clinically relevant deviations. The optimal sensitivity of a fingerprint can only be estimated prior to data analysis, but may be refined retrospectively through methodological adjustments informed by the results. In the present fingerprint, discarding one hemisphere could increase sensitivity but would entail a loss of information given that language plasticity can occur bilaterally (Deverdun et al., 2020; Duffau, 2014). A more promising refinement may therefore be to employ block-restricted permutations (Winkler et al., 2014) that shuffle connectivity only within hemispheres rather than across all targets. Such an approach would preserve large-scale asymmetry in the null distribution, providing a more stringent test and thereby improving sensitivity for clinically meaningful deviations Naturally, the dependency on target composition also has implications for the exclusion of lesion-affected targets. While supplementary analyses demonstrated that such exclusion did not systematically alter fingerprint classification, it nonetheless modifies fingerprint configuration — and does so unevenly: For example, removing a target that is both boundary-defining and distant from the next closest value will disproportionately shift the normalization range, whereas removing a target that lies close to neighboring values will have a comparatively minor effect. In the present study, no systematic pattern was observed in which fingerprints with specific excluded targets were more likely to appear atypical than others. Nevertheless, this represents an inherent methodological challenge for fingerprint analyses in brain tumor populations, where lesion location varies across patients and inevitably leads to different targets being excluded. Optimizing the handling of lesion-affected targets and systematically evaluating ROI combinations with respect to their sensitivity characteristics remain important methodological questions for future research.

A second aspect highlighted by the case example concerns the use of normalized versus absolute connectivity values. While MD and binary classifications are derived from normalized fingerprints, absolute values have likewise been shown to provide clinically relevant insights (Sun et al., 2024). Fig. 4 illustrates that the normalized fingerprints do not capture the markedly reduced preoperative connectivity, which increased postoperatively. For clinical applications, we therefore argue that fingerprints should be visualized using absolute values to avoid overlooking such changes, given that information about network structure is already inherently represented by MD and the (a)typical classification.

Beyond methodological considerations, group-level comparisons revealed differences in network deviation and language performance between high- (HGG) and low-grade gliomas (LGG). Previous studies have consistently reported more severe and widespread network disruption in HGG, often attributed to their aggressive and infiltrative growth (Derks et al., 2019; Hart et al., 2019; Yuan et al., 2020). In the present cohort, this distinction did not reach significance preoperatively but an effect emerged in the immediate postoperative phase, where HGG patients showed greater deviations from the typical network structure, paralleled by stronger declines in verbal fluency, language production, and written language. Given the limited group sizes, nonparametric analyses were conducted, preventing statistical control for age; thus, age should be considered a potential contributor to the observed group difference, as HGG patients were older than those with LGG. Beyond age, several other factors are likely to contribute to the more pronounced alterations observed in HGG, including a greater extent of resection (Hervey-Jumper and Berger, 2016) and rapid tumor progression, which may restrict preoperative plasticity and thereby might make acute postoperative remapping more critical for sustaining function (Barcia et al., 2012; Duffau, 2014, 2017). While the reported findings can be situated within the existing literature, they should be interpreted as exploratory given the limited group size. Notably, in HGG, a rightward shift in connectivity directly after surgery predicted smaller declines in phonological fluency; an effect not observed in the combined sample. Given the small subgroup size, this finding must be interpreted with considerable caution, as small samples increase the risk of unstable estimates. Should this pattern be confirmed in adequately powered samples, it may be interpreted within hierarchical compensation models, which propose contralateral recruitment once ipsilateral mechanisms are no longer sufficient (Cargnelutti et al., 2020; Nieberlein et al., 2023). This would be in line with the more pronounced postoperative deficits in HGG versus LGG, suggesting that greater left-hemispheric disruption may have necessitated contralateral support. Prior research has provided evidence that the adaptive involvement of the right hemisphere in language processing may be temporally contingent: while early recruitment has been shown to support acute compensation, persistent right-hemisphere dominance in the chronic stage has been linked to poorer outcomes, presumably reflecting a less efficient reorganization process (Hartwigsen et al., 2020; Turkeltaub et al., 2011; Zimmermann et al., 2020). Conclusions about changes in the (mal)adaptivity of right-hemisphere recruitment from postoperative to follow-up assessment cannot be drawn from our data. Descriptively, however, in our cohort, in the absence of tumor progression, connectivity shifted further toward the left hemisphere in all patients, accompanied by stable or improved language performance. If corroborated statistically in larger samples, this pattern could suggest a favorable neural response associated with reestablishing or strengthening left-hemispheric dominance beyond the immediate postoperative period. Further investigation is required before firm conclusions can be drawn.

The findings of the present study underscore the limitations of group-based approaches and highlight the potential of connectivity fingerprinting to delineate patient-specific network patterns and advance personalized medicine (Whitcomb, 2012). The considerable variability in functional reorganization trajectories across patients implies that at group-level consistent patterns are (1) difficult to detect, and (2) even if common patterns were to emerge, they do not permit deductive inference at the individual level. Thus, while scientifically valuable, such findings offer limited predictive value for individual outcomes. This challenge is amplified in longitudinal designs, where unpredictable disease progression and treatment adaptations introduce increasing variability over time, complicating group-level analyses. Yet efforts to maintain sample homogeneity by excluding or stratifying patients based on clinical factors stand at odds with the goal of creating prognostic models applicable to all patients, irrespective of pathology or disease course. The single-subject approach circumvents these problems by enabling the analysis of each patient's trajectory independently, without blurring interindividual nuances. Clinically, the reliance on resting-state fMRI makes fingerprinting feasible even in patients with limited task capacity, which broadens its applicability in clinical practice. (Kumar et al., 2020). The potential of fingerprinting extends beyond neurosurgical contexts, including psychiatric and neurological disorders, for example as a tool to support (differential) diagnosis (Balsters et al., 2018; Ichikawa et al., 2020; Morgan et al., 2022; Stampacchia et al., 2024).

The present findings should be interpreted in light of several limitations, which point toward important directions for future work. First, only twelve datasets were available at the three-month follow-up, constraining statistical power and limiting the generalizability of longitudinal findings. Second, several factors inherent to fMRI in brain tumor patients warrant consideration. While seed signal quality (tSNR) was not related to MD, effects of neurovascular uncoupling, a known limitation of fMRI in brain tumor patients, cannot be fully ruled out. Future studies incorporating cerebrovascular reactivity mapping may allow a more direct assessment of neurovascular uncoupling and help to account for this potential confound. Furthermore, in some patients lesions were located in or near the seed region; although seed–tumor overlap was not associated with reduced seed tSNR, potential perilesional effects cannot be entirely excluded. Finally, exact voxel-level correspondence of ROIs across time points cannot be guaranteed, as direct longitudinal registration was avoided due to tumor mass effect and surgical changes. Third, the healthy control group was not age-matched to the patient cohort. While normalization to a 0–1 range may partially attenuate global age-related differences in connectivity strength, potential influences on the relative weighting of individual connections cannot be ruled out. Future studies should include control groups that are matched for age and, ideally, for other demographic variables as well, in order to further strengthen the normative reference model. Fourth, it should be noted that tumor-induced functional reorganization can result in language-critical processing being shifted away from the anatomical IFG in some patients, such that the atlas-based seed no longer coincides with the functional hub of the language network. This is an inherent limitation of any atlas-based seed definition in the presence of cortical plasticity and remains an important consideration for individual-level interpretation and future study design. A functionally defined seed based on individual task-based activation could in principle better capture the actual locus of language processing. However, this would require an additional task-based fMRI acquisition and would introduce further inter-patient variability into the analysis, which, while desirable from an individualization perspective, complicates comparability. More broadly, a systematic comparison of multiple seed definitions, including diverse atlas-based as well as functionally defined seeds and their respective associations with language outcomes would substantially strengthen the methodological foundation of connectivity fingerprinting and represents a key direction for future work. Fifth, we deliberately included a heterogeneous patient cohort with the primary aim of capturing individual network alterations and their relationship to language changes at the single-patient level, independent of pathological characteristics such as tumor grade (Voets et al., 2021). Given this heterogeneity, the present analyses primarily capture systems-level deviations in network organization in relation to language performance, whereas more specific mechanistic interpretations of the observed alterations remain constrained. Accordingly, the influence of factors such as prior surgery or peri-operative conditions could only be explored to a limited extent. This heterogeneity implies that fingerprint interpretation at the single-patient level requires validation in larger cohorts. Establishing normative datasets for clinically defined subgroups will therefore be essential to contextualize individual fingerprints and enhance their diagnostic and prognostic utility. Future research should further aim to disentangle the influence of distinct clinical factors on connectivity fingerprints, to systematically evaluate alternative seed definitions and ROI configurations, and to identify markers of (mal)adaptive fingerprint patterns. Computational approaches, including AI-based methods, may help to detect subtle features that precede or accompany changes in language performance, ultimately enhancing the prediction of individual trajectories, informing disease monitoring, and guiding targeted interventions.

### 4.1 Concluding remarks

Resting-state connectivity fingerprints offer a promising approach for characterizing alterations in language networks in patients with brain tumors. Although continued methodological refinement is needed at present, their integration into clinical workflows opens new perspectives for individualized, course-oriented patient care. With further validation in larger cohorts, fingerprinting holds promise for advancing personalized medicine in neuro-oncology, with potential applicability to other neurological and psychiatric conditions.

## Declaration of generative AI and AI-assisted technologies in the writing process

During the preparation of this work the authors used ChatGPT (OpenAI, San Francisco, CA, USA) in order to improve readability and language. After using this tool, the authors reviewed and edited the content as needed and take full responsibility for the content of the published article.

## Funding

This study was conducted within the project *“Augmented Reality* supported *Functional Brain Mapping for Navigated Surgery Preparation and Education* *(nARvibrain)”*, funded by the 10.13039/501100004955Austrian Research Promotion Agency (10.13039/501100004955FFG) [Project No. 894756].

## CRediT authorship contribution statement

**Pia Ritter:** Conceptualization, Data curation, Formal analysis, Investigation, Methodology, Visualization, Writing – original draft. **Manuela Christine Michenthaler:** Conceptualization, Data curation, Project administration, Resources, Writing – review & editing. **Karla Zaar:** Conceptualization, Data curation, Project administration, Resources, Writing – review & editing. **Kariem Mahdy Ali:** Conceptualization, Funding acquisition, Project administration, Resources, Writing – review & editing. **Gernot Reishofer:** Conceptualization, Funding acquisition, Writing – review & editing. **Stefan Wolfsberger:** Conceptualization, Resources, Supervision, Writing – review & editing. **Hannes Deutschmann:** Conceptualization, Resources, Supervision, Writing – review & editing. **Margit Jehna:** Conceptualization, Data curation, Methodology, Project administration, Resources, Supervision, Writing – review & editing.

## Declaration of competing interest

The authors declare that they have no known competing financial interests or personal relationships that could have appeared to influence the work reported in this paper.

## Data Availability

Data are not publicly available due to privacy and ethical restrictions but may be shared in de-identified form upon reasonable request and subject to appropriate ethical approval.
