# Supplementary material for: Identification of abnormal neural language networks by reading “brainprints” in patients with brain tumors
Source: Neuroimage Rep. 2026 Jun 20;6(3):100374. doi: 10.1016/j.ynirp.2026.100374 (PMC13314782; doi:10.1016/j.ynirp.2026.100374)
Supplement: Multimedia component 1 [file mmc1.docx]

**Supplementary Methods**

*Functional MRI Sequence Parameters*

MRI data were acquired on a 3T Siemens Prisma_fit scanner (Siemens Healthineers, Erlangen, Germany) equipped with syngo MR E11 software. For Functional MRI (BOLD EPI) a multiband echo-planar imaging sequence was used with the following parameters: repetition time (TR) = 933 ms, echo time (TE) = 33.4 ms, flip angle = 64°, matrix size = 96 × 96, field of view (FOV) = 192 × 192 mm², voxel size = 2 × 2 × 2 mm³, slice thickness = 2 mm, spacing between slices = 1.999 mm, 96 slices acquired in interleaved order, patient position = head-first supine (HFS). The acquisition yielded 338 volumes, corresponding to a total acquisition time of ~5.3 min. Participants were instructed to close their eyes during acquisition. A sagittal 3D MPRAGE sequence was acquired with TR = 1900 ms, TE = 2.22 ms, flip angle = 9°, voxel size = 1 × 1 × 1 mm³, matrix size = 256 × 256, 256 slices, FOV = 256 × 256 mm². A transverse TIRM sequence was acquired with TR = 8300 ms, TE = 78 ms, flip angle = 150°, slice thickness = 2 mm, in-plane resolution = 0.98 × 0.98 mm², matrix size = 256 × 256, 192 slices, FOV = 250 × 250 mm².

*Selective Target Removal in Fingerprints*

The minimal number of voxels required to make valid inferences about an entire region was calculated using the formula $n=\frac{Z^{2}\cdot p(1-p)}{E^{2}}$ (Israel, 1992). Here, $Z$ is the z-value corresponding to the chosen confidence level (1.96 for 95%), $E$is the margin of error (0.05), and $p$ represents the proportion of voxels considered representative, with $p=0.5$ reflecting maximum uncertainty. For our analysis, we used $p=0.8$, since HCP-MMP1.0 regions (Glasser et al., 2016) are defined by functional, anatomical, and cytoarchitectonic boundaries, allowing us to assume relative homogeneity within each region. Thus, at least 80% of voxels are considered representative. The finite population correction (FPC) was then applied to account for the limited number of voxels per region: $n_{adj}=\frac{n}{1+\frac{n-1}{N}},$ where $N$ denotes the total number of voxels, determined from the average region sizes of the control group. This adjustment specifies the required number of non-overlapping voxels in a finite population to ensure valid statements about the entire region at 95% confidence. If too few voxels remain after removing overlapping voxels, the entire region is to be excluded from the analysis. For the seed region, this would have led to the exclusion of the patient, as seed-based correlation (SCA) results would have been considered unrepresentative of the entire region; however, this was not the case.

To test whether target exclusion alters fingerprint configuration sufficiently to bias classifications toward atypical, we performed a control analysis. We identified 17 unique target exclusion patterns in patients with atypical fingerprints (e.g., PSL+STSdp, AVI , AVI+55b, PSL etc.). Each pattern was then applied to a randomly selected patient with a typical fingerprint and no tumor-related target exclusions (n=18 available). For patterns that occurred multiple times in the atypical group, different typical patients were randomly selected for each application. Fingerprint classifications were subsequently recalculated based on the reduced target set. All resulting fingerprints remained classified as typical, indicating that while target exclusion alters fingerprint configuration, this effect alone does not systematically shift classifications from typical to atypical.

**Supplementary Tables**

*Supplementary Table S.1*

The table below provides an overview of the functional role of the selected regions of interest (ROIs), with primary reference to Rolls et al. (2022) and additional information drawn from the supplementary materials of Glasser et al. (2016) and Price (2012). Rolls et al. (2022) used the HCP-MMP1.0 atlas to examine the resting-state functional connectivity of cortical areas in over 1,000 participants from the Human Connectome Project (HCP). Based on this analysis, six large-scale cortical networks were identified, including three networks relevant to language processing: a frontal network primarily associated with language production and syntactic processing (Group 2; TGv, 44, 45, 47l, SFL und 55b), a superior temporal network involved in auditory, prosodic, and body-related semantic processing (Group 3;  **A5, STGa, STSda, STSdp, PSL, STV, TPOJ1**), and an inferior temporal semantic network that integrates visual meaning, memory, and reward processing (Group 1; STSvp, STSva, TE1a, TGd, PGi, prefrontal regions). The table below focuses exclusively on the regions used in the present study and therefore does not encompass the full set of findings reported by Rolls et al. (2022).

The selected ROIs are regions identified by Rolls et al. (2022) as belonging to, or interacting with, IFG-associated language subnetworks involving areas 44, 45, and 47l. The selected regions do not represent all regions showing connectivity with IFG-related language areas, but rather a theoretically and methodologically motivated subset of nodes drawn from all three major subnetworks described by Rolls et al. (2022), with the goal of capturing distributed frontal, temporal, and parietal components of the left-hemispheric language network. AVI is the only target region not identified by Rolls et al. (2022) as showing strong connectivity with IFG-related language areas. Its inclusion was motivated by frequent co-activation in task-based fMRI paradigms at our institution (unpublished data) and because its expected comparatively lower connectivity with the IFG was anticipated to contribute to the topographic variability of the fingerprint profile. Because the fingerprint is normalized, target composition cannot be determined solely on the basis of linguistic relevance, but must also account for the need to preserve sufficient variability across target regions during normalization. This methodological consideration is discussed in detail in the Discussion. Accordingly, the present fingerprint represents a theoretically and methodologically motivated subset of language-relevant regions within the Glasser et al. (2016) atlas rather than a comprehensive mapping of the full language network, and its properties are inherently tied to this specific selection.

| **Abbre-vation** | **Full Region Name** | **Functional Group** *(Information from Rolls et al., 2022)* | **Additional Information** |
| --- | --- | --- | --- |
| **SFL** | Superior Frontal Language Area | - Group 2; strong effective connectivity with areas 44/45 (putative output region);receives input from semantic groups 1 & 3 - Potential supplementary premotor area involved in speech production | Left hemisphere more active during language tasks; right during social cognition (ToM) (supplementary materials Glasser et al., 2016) |
| **PGi** | Inferior Parietal Gyrus | - Group 1; provides output to areas 44/45 - Involved in object-related semantic processing and episodic contextualization - Part of angular gyrus together with PGs; PGi is more involved in language processing than PGs |  |
| **TGv** | Ventral Temporal Gyrus | - Group 2; temporal component of group 2 network; effective connectivity with 44, 45 and 47l - output to semantic areas from group 1; input from semantic regions (TGd, STSvp) - Likely involved in processing high-level abstract semantic concepts and transmitting them to syntactic/motor language regions |  |
| **PSL** | Perisylvian Language Area | - Group 3; output to and, to a lesser extent, input from areas 44/45/55b - Part of strongly left-lateralized language network - Processes semantic information from auditory and body-related inputs (e.g. prosody, movement…) | Involved in speech comprehension and phonological processing; part of classical Wernicke’s area; not sig. activated by non-linguistic tasks (supplementary materials Glasser et al., 2016) |
| **55b** | Area 55b | - Group 2; robust effective connectivity with 44/45 (putative output area) - language-related premotor area - receives input from various language-related areas (e.g. 44/45/47l/Tgv/PSL/STSdp); output to STSdp, PSL etc. - likely involved in planning and execution of articulatory processes | One of the most strongly activated regions in the language network; key hub with strong left-lateralization (supplementary materials Glasser et al., 2016) |
| **AVI** | Anterior Ventral Insular Area | - Not part of the three language-related networks defined by Rolls et al. (2022) | Frequently co-activated in our task-based fMRI paradigms (unpublished data).  increased activation in response to complex or novel subsyllabic verbal sequences in left anterior insula; Thought to be involved in motor execution and coordination of articulatory and phonatory muscles  (Price, 2012)  Strongly activated during emotion tasks (interoceptive and salience-related processes) (supplementary materials Glasser et al., 2016) |
| **STSdp** | Superior Temporal Sulcus, dorsal posterior | - Group 3; Robust effective connectivity with area 44 and 45 (putative output area). - Input from auditory and visual sensory areas - central multimodal hub for linguistic and socio-communicative processing, providing input to language production areas; integrates auditory and visual signals (e.g., speech sounds and lip movements) |  |
| **44** | Area 44 (part of Broca’s Area) | - Group 2; input from semantic regions in groups 1 and 3, including TGv; Output to areas 55b, SFL etc. - Supports language production, particularly syntactic structuring and articulation; integrates semantic input into motor speech plans |  |
| **45** | Area 45 (part of Broca’s Area) | - Group 2; input from semantic regions in Group 1 (e.g. PGi) and superior STS-regions from group 3 (e.g. STSdp and PSL) as well as TGv - Output to 55b/SFL/STSdp/PSL and more - Receives input from high-level semantic areas; Semantically more engaged than Area 44 |  |
| **47l** | Area 47l (lateral) | - Group 2; input from semantic regions in group 1 (e.g. PGi) and also from group 2 itself; output to sreas 44/45/55b/SFL/ temporal regions and prefrontal cortex - Involved in semantic aspects of language production, especially contextual integration - May contribute to motor preparation via connections to 55b and SFL |  |

Supplementary Table S.2. *Descriptive statistics of Manhattan Distance (MD) and language performance variables across three time points for the patient group.*

| Measure | Pre *M* ± *SD* | Post *M* ± *SD* | 3M FU *M* ± *SD* | Sig. changes |
| --- | --- | --- | --- | --- |
| Manhattan Distance | 1.15 (.28) | 1.51 (.29) | 1.49 (.63) |  |
| Semantic fluency | -.36 (.15) | -.99 (.16) | -.58 (.37) | Pre > Post**, Pre > 3M* |
| Phonological fluency | -.48 (.13) | -.95 (0.12) | -.55 (.22) | Pre > Post** |
|  | *Mdn* [IQR] | *Mdn* [IQR] | *Mdn* [IQR] | Sig. changes |
| Language production | 99.0 (1.5) | 95.25 (42.88) | 98.00 (23.75) | Pre > Post** |
| Language comprehension | 100 (0.0) | 97.0 (13.13) | 100 (16.75) | Pre > Post** |
| Written language | 98.66 (1.17) | 88.33 (20.58) | 95.66 (13.17) | Pre > Post** |

*Note.* Manhattan Distance (MD) is reported as a z-score standardized against the control group (*M* ± *SD*). Values for semantic and phonological fluency are reported as z-scores standardized against the healthy control group and are given as *M* ± *SD*. AAT subtests are reported in percentile ranks (PRs) and presented as Median [IQR]. Results refer to patients only, not controls. ***p* < .01, **p* < .05. Pre = preoperative time point; Post = postoperative time point; 3M FU = three-months follow-up.

Supplementary Table S.3. *Linear mixed model results from sensitivity analyses restricted to primary CNS tumors (excluding metastasis cases) with Manhattan Distance (MD) as dependent variable.*

| Predictor | *F*(df) | *p* | *η²ₚ* |
| --- | --- | --- | --- |
| Time point | *F*(2,41.22) = 0.45 | .450 | 0.02 |
| Language production | *F(*1,28.44) = 5.39 | .028* | 0.16 |
| Language comprehension | *F(*1,47.58) = 6.38 | .015* | 0.12 |
| Written language | *F(*1,34.55) = 3.41 | .073 | 0.09 |
| Semantic fluency | *F(*1,27.39) = 3.30 | .080 | 0.11 |
| Phonological fluency | *F(*1,46.69) = 0.04 | .833 | 0.001 |

*Note.* All results are age-controlled. Linear Mixed Models (LMM) were conducted with Manhattan Distance (MD) as dependent variable; means represent estimated marginal means. Partial eta squared (η²ₚ) was calculated from the F statistics using η²ₚ = F / (F + df_error). **p* < .05, ***p* < .01

Supplementary Table S.4. *Manhattan Distance (MD) values for each patient across time points.*

| Patient | Pre | Post | 3M FU | Δ MD (pre→post) | Δ MD (post→3M) | Excluded Targets |
| --- | --- | --- | --- | --- | --- | --- |
| 1 | 0.5035 | 0.6356 |  | 0.1321 |  | None |
| 2 | 0.8224 | 0.8354 |  | 0.013 |  | STSdp, PSL |
| 3 | 2.2014 | 2.5413 | 1.7890 | 0.3399 | -0.7523 | STSdp, PSL |
| 4 | 2.5607 | 2.3035 |  | -0.2572 |  | TGv |
| 5 | 1.1907 | 0.3287 | -0.8237 | -0.862 | -1.1524 | None |
| 6 | -0.4583 | 4.6759 |  | 5.1342 |  | PSL |
| 7 | 0.2828 | 1.1899 | 0.2847 | 0.9071 | -0.9052 | None |
| 8 | 1.4704 | 1.5687 |  | 0.0983 |  | AVI |
| 9 | 2.6500 | 2.4559 | -0.8571 | -0.1941 | -3.313 | STSdp, PSL |
| 10 | -0.5651 | -0.4121 |  | 0.153 |  | TGv, STSdp |
| 11 | 1.8317 | 1.0870 |  | -0.7447 |  | 55b, SFL |
| 12 | -0.8096 | 0.6610 |  | 1.4706 |  | TGv |
| 13 | 0.7148 | 1.0361 |  | 0.3213 |  | AVI, STSdp |
| 14 | 1.5972 | 2.8431 |  | 1.2459 |  | TGv, STSdp |
| 15 | -0.1923 | -0.9227 |  | -0.7304 |  | None |
| 16 | -0.0968 | 1.3170 |  | 1.4138 |  | None |
| 17 | 4.3714 | 3.8405 | 3.1389 | -0.5309 | -0.7016 | 55b, AVI |
| 18 | 1.9816 |  | -0.0966 |  |  | STSdp |
| 19 | 1.7996 | 1.2282 | 3.2845 | -0.5714 | 2.0563 | None |
| 20 | 2.0405 | 3.6612 |  | 1.6207 |  | AVI |
| 21 | 2.8353 | 2.7783 |  | -0.057 |  | STSdp, PSL |
| 22 | 0.0819 | -1.1641 | 4.1169 | -1.246 | 5.281 | STSdp, PSL |
| 23 | 4.6297 | 2.4472 | 0.2871 | -2.1825 | -2.1601 | PSL |
| 24 | -0.9679 | 1.5124 | 0.3610 | 2.4803 | -1.1514 | None |
| 25 | 0.7166 | 0.3123 | 0.3662 | -0.4043 | 0.0539 | AVI |
| 26 | -1.0036 |  | 6.1057 |  |  | TGv |
| 27 | 0.9592 | 1.0266 |  | 0.0674 |  | None |

*Note.* Patient indices correspond to those used in Figure 3. Manhattan Distance (MD) values are expressed as z-scores relative to the healthy control group. Pre = preoperative time point; Post = postoperative time point; 3M FU = three-month follow-up. ΔMD (pre→post) indicates the change from preoperative to postoperative values (post minus pre), and ΔMD (post→3M) indicates the change from postoperative to three-month follow-up values (3M minus post). Higher ΔMD values indicate greater deviation at the later time point compared to the earlier time point.

Supplementary Table S.5. *Associations between data quality metrics and Manhattan Distance (MD).*

| Predictor | *F* | *p* |
| --- | --- | --- |
| Seed-region tSNR | *F*(1, 50.10) = 0.03 | .866 |
| Mean ROI tSNR | *F*(1, 51.24) = 1.12 | .294 |
| Mean FD | *F*(1, 48.29) = 1.48 | .230 |

*Note.* Linear Mixed Models (LMM) with Manhattan Distance as dependent variable; tSNR = temporal signal-to-noise ratio; ROI = Region of interest; FD = framewise displacement.

Supplementary Table S.6. *Associations between binary fingerprint classification (typical vs. atypical) and data quality metrics.*

| Outcome | *F* | *p* |
| --- | --- | --- |
| Seed-region tSNR | *F*(1, 34.36) = 0.84 | .365 |
| Mean ROI tSNR | *F*(1, 31.14) = 0.05 | .818 |
| Mean FD | *F*(1, 43.45) = 0.00 | .953 |

*Note.* Linear Mixed Models (LMM) with binary fingerprint classification as independent variable; tSNR = temporal signal-to-noise ratio; ROI = Region of interest; FD = framewise displacement.

Supplementary Table S.7. *Distribution of excluded target combinations across fingerprint classifications.*

| Excluded target(s) | Typical fingerprints | Atypical fingerprints |
| --- | --- | --- |
| STSdp + PSL | 7 | 6 |
| STSdp + TGv | 3 | 1 |
| SFL + 55b | 2 | 0 |
| AVI + STSdp | 1 | 1 |
| 55b + AVI | 0 | 3 |
| STSdp only | 2 | 0 |
| TGv only | 3 | 3 |
| PSL only | 2 | 3 |
| AVI only | 3 | 4 |

Supplementary Table S.8. *Statistical results of sensitivity regression analyses restricted to primary CNS tumors (excluding metastasis cases) with Manhattan Distance (MD) as predictor.*

| Time point | Fluency Subtest | *F*(df) | *p* | *R²* (adj.) | β | *t* | *p* (β) |
| --- | --- | --- | --- | --- | --- | --- | --- |
| Pre | Phonological | *F*(2,20) = 0.82 | .456 | -.017 | -0.27 | -1.27 | .218 |
|  | Semantic | *F*(2,20) = 0.13 | .878 | -0.86 | -0.11 | -0.50 | .625 |
| Post | Phonological | *F*(2,18) = 5.78 | .011 | .324 | -0.62 | -2.69 | .015* |
|  | Semantic | *F*(2,18) = 0.57 | .577 | -.05 | -0.28 | -0.97 | .347 |

*Note.* All results are age-controlled. Regression analyses were conducted with Manhattan Distance (MD) as predictor. **p* < .05, ***p* < .01. Pre = preoperative time point; Post = postoperative time point.

Supplementary Table S.9. *Analyses examining potential effects of seed–tumor overlap on Manhattan Distance (MD).*

| Analysis | Test statistic | *p* | Effect size |
| --- | --- | --- | --- |
| overlap (yes vs. no) | *F*(1, 52.35) = 0.28 | .596 | *η²ₚ* = .005 |
| Overlap % vs MD (pre) | *ρ* = −.16 | .417 |  |
| Overlap % vs MD (post) | *ρ* = −.26 | .315 |  |
| Overlap % vs MD (3M FU) | *ρ* = .16 | .627 |  |

*Note*. Linear Mixed Models (LMM) were used to test whether seed–tumor overlap predicted Manhattan Distance (MD), with time point included as a repeated factor and subject as a random effect. Overlap was examined as a binary variable. Spearman correlations assessed associations between continuous overlap percentage and MD at each time point. Pre = preoperative time point; Post = postoperative time point; 3M FU = three-months follow-up.

Supplementary Table S.10. *Results of analyses testing clinical variables as predictors of Manhattan Distance.*

| Measure | Time point | Test statistic | *p* | Effect size |
| --- | --- | --- | --- | --- |
| Recurrence status |  | *F*(1, 29.18) = 0.63 | .435 | *η²ₚ* = .02 |
| Tumor volume | Pre | *t*(25) = 1.03 | .31 | *R*² = .04 |
|  | Post | *t*(23) = 0.65 | .52 | *R*² = .02 |
|  | 3M FU | *t*(10) = −0.49 | .64 | *R*² = .02 |
| Tumor location | Pre | *H*(3) = 0.67 | .88 | ε² = .01 |
|  | Post | *H*(3) = 1.61 | .66 | ε² = .03 |

*Note****.*** Linear mixed-effects models were used to test whether recurrence status predicted Manhattan Distance (MD). Tumor location (frontal, temporal, parietal, insula-involving) was analyzed separately at each time point using Kruskal–Wallis tests due to small subgroup sizes. Tumor volume as predictor of MD was analyzed using linear regression at each time point. Effect sizes are reported as partial η² for mixed models, ε² for Kruskal–Wallis tests, and *R*² for linear models. Pre = preoperative time point; Post = postoperative time point; 3M FU = three-months follow-up.

**Supplementary Figures**

*
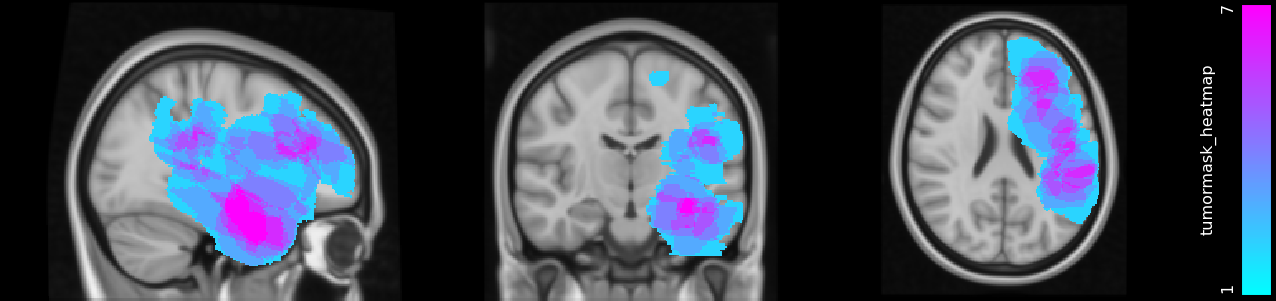
Supplementary Figure S.1*


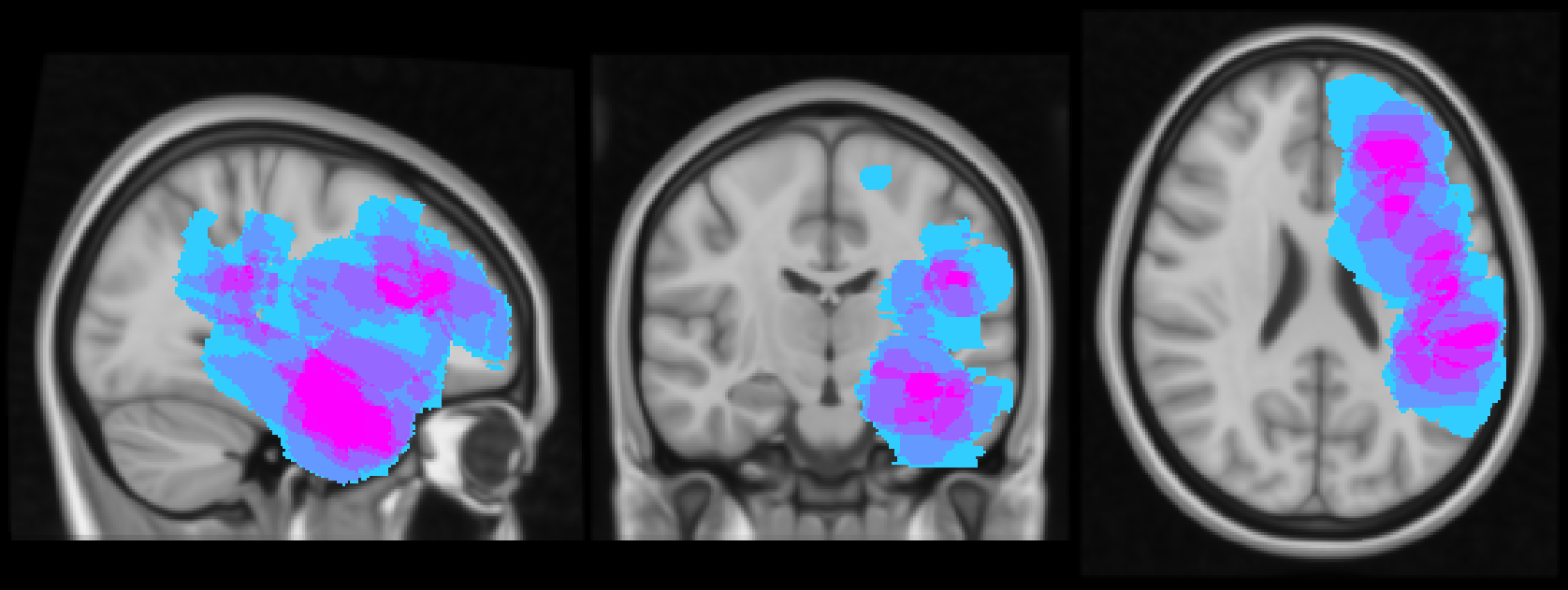


**Figure S.1.** Heatmap of tumor masks (at preoperative time point) for all patients.

*Supplementary Figure S.2*

1. *
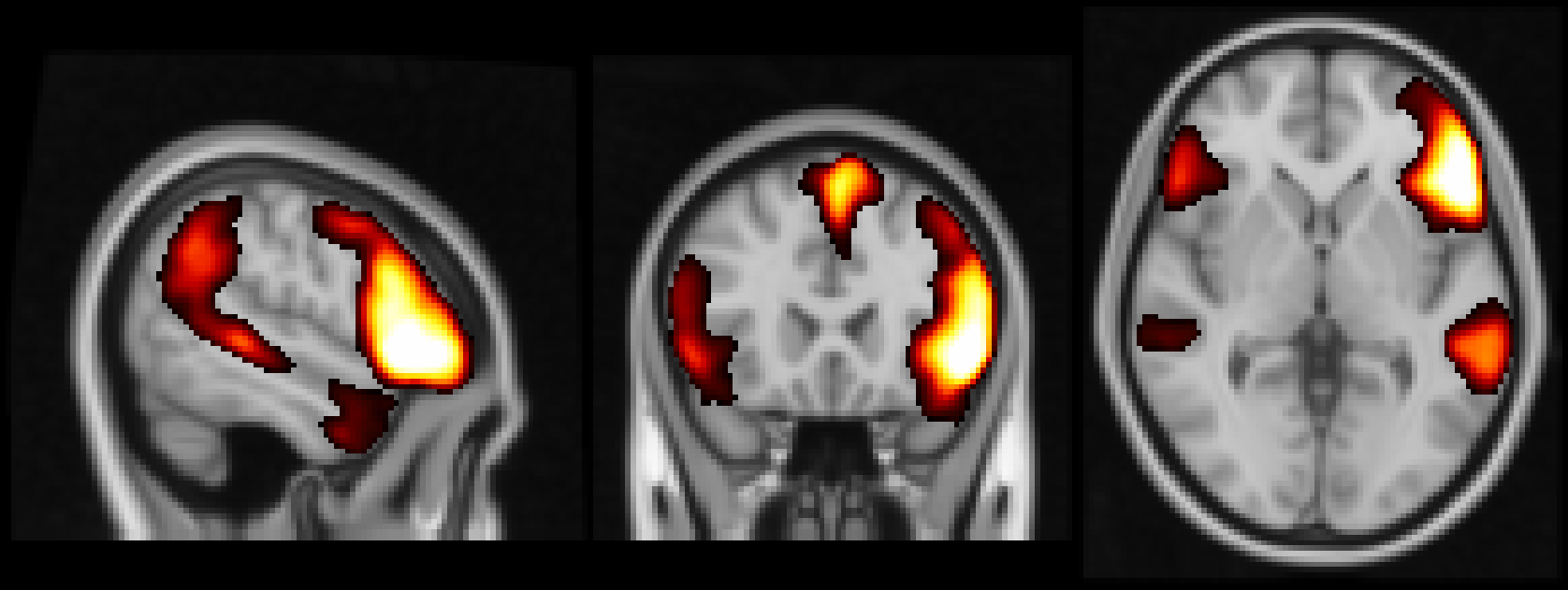
*
2. *
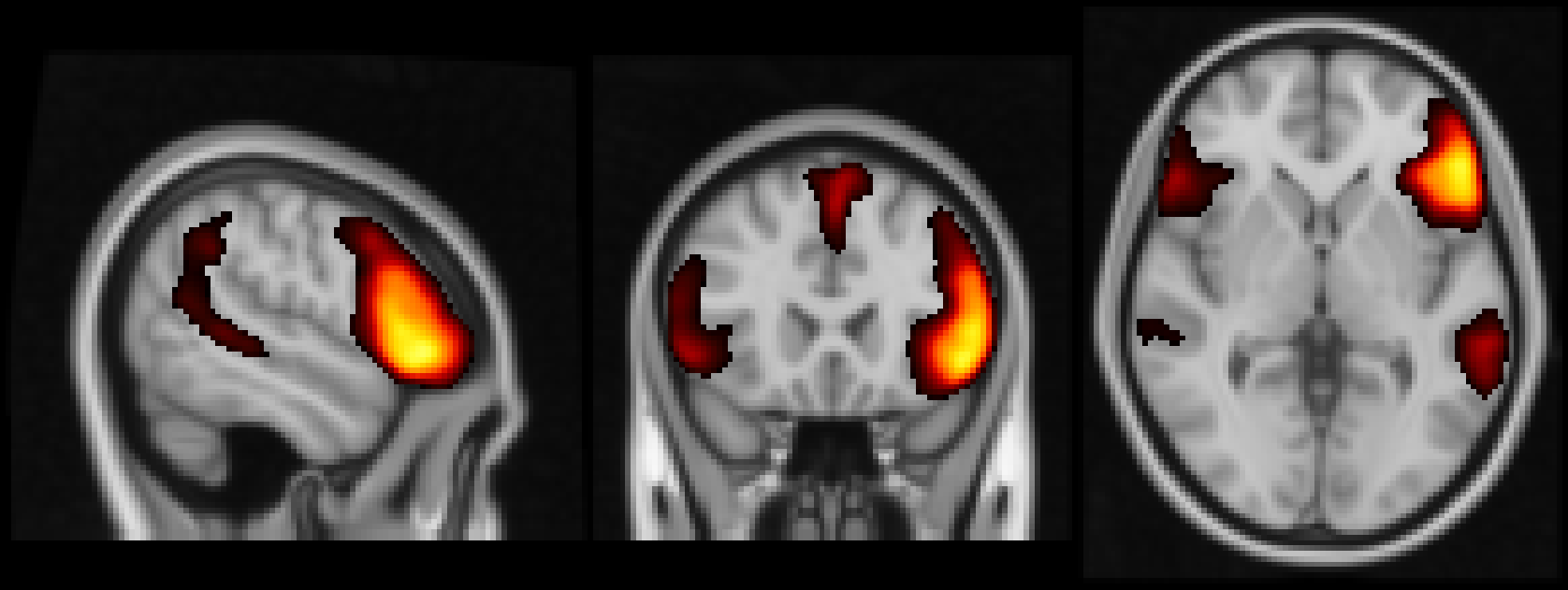
*

**Figure S.2.** Mean SCA maps across all time points: (a) patients and (b) controls. Both maps are thresholded at identical Fisher z-transformed correlation values (min 0.23, max 0.7).

*Supplementary Figure S.3*

*
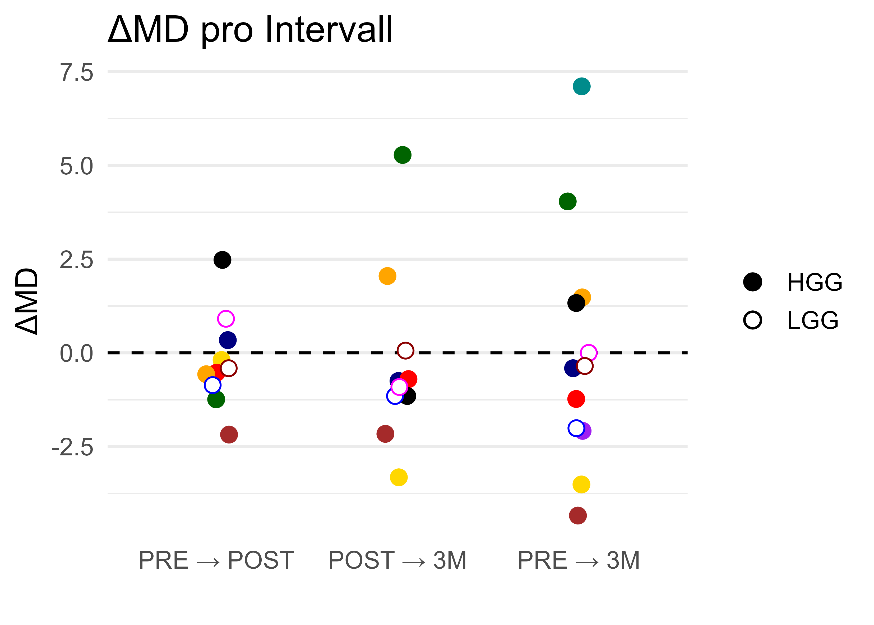
*

**Figure S.3.** Individual changes in Manhattan Distance (MD) across time points for all patients with available three-month follow-up data. The figure shows change scores (ΔMD) for each patient between the preoperative and postoperative assessment (pre→post), between postoperative and three-month follow-up (post→3M), and between preoperative and three-month follow-up (pre→3M). Each colored dot represents one patient (same color indicates the same patient across comparisons). Positive values indicate an increase in deviation from the healthy reference network over time, whereas negative values indicate a decrease in deviation (i.e., movement toward the healthy reference pattern). The dashed horizontal line marks no change (ΔMD = 0). Change scores involving the three-month follow-up show a wider spread of values across patients than the pre→post comparison, reflecting more heterogeneous longitudinal trajectories.

*Supplementary Figure S.4*

**Figure S.4.** (a) Original seed (red) and trimmed seed (green) in an exemplary patient (Pat. A), corresponding to the fingerprint shown at the top left in (b). (b) Six fingerprints from different patients before (red) and after (gray) seed size reduction. Fingerprint patterns remained nearly identical after reduction.


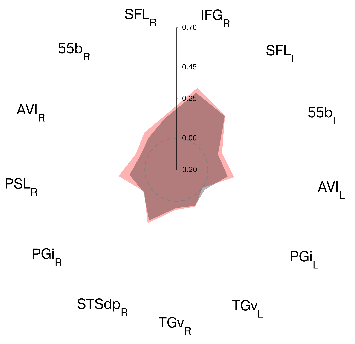

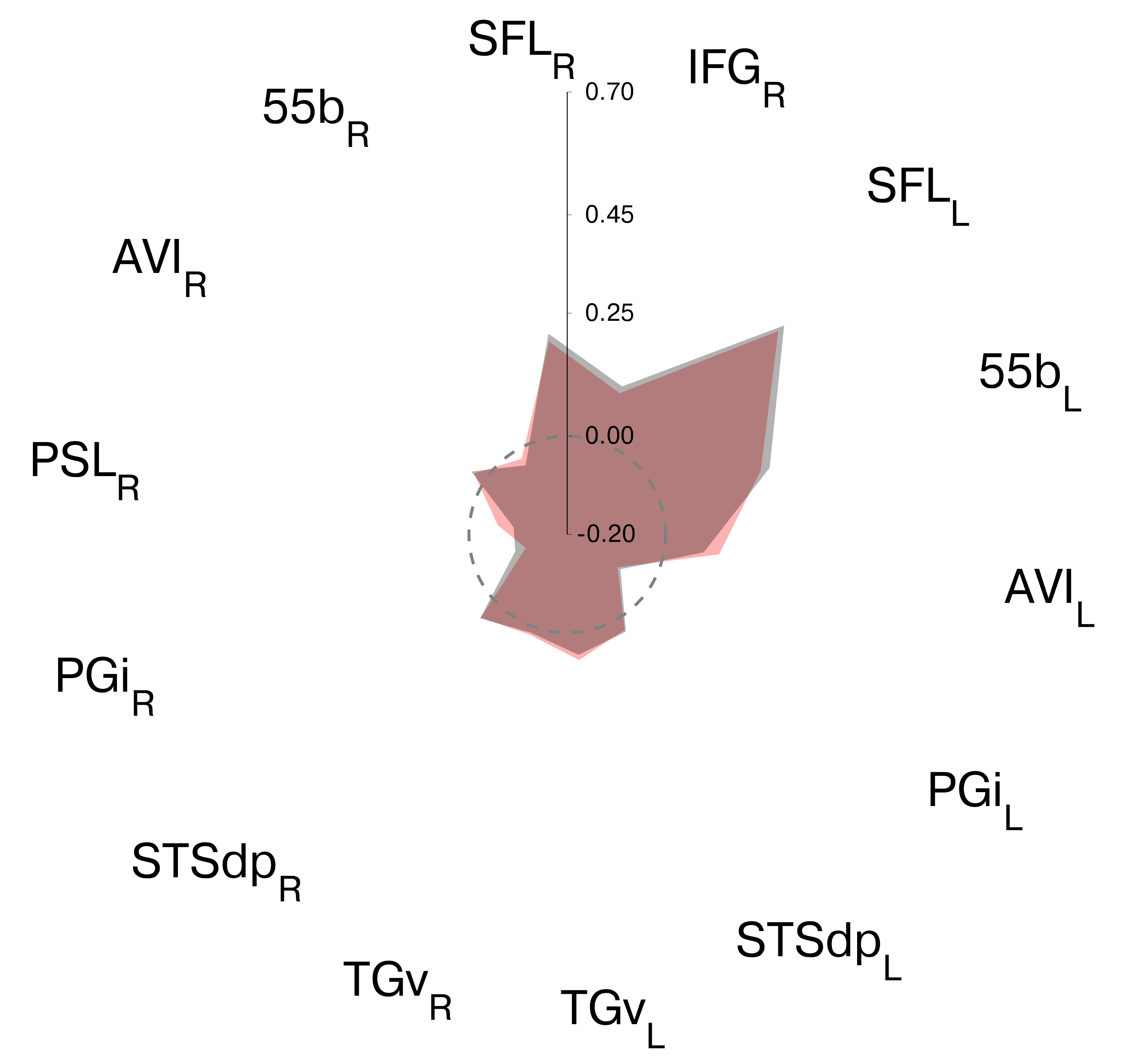

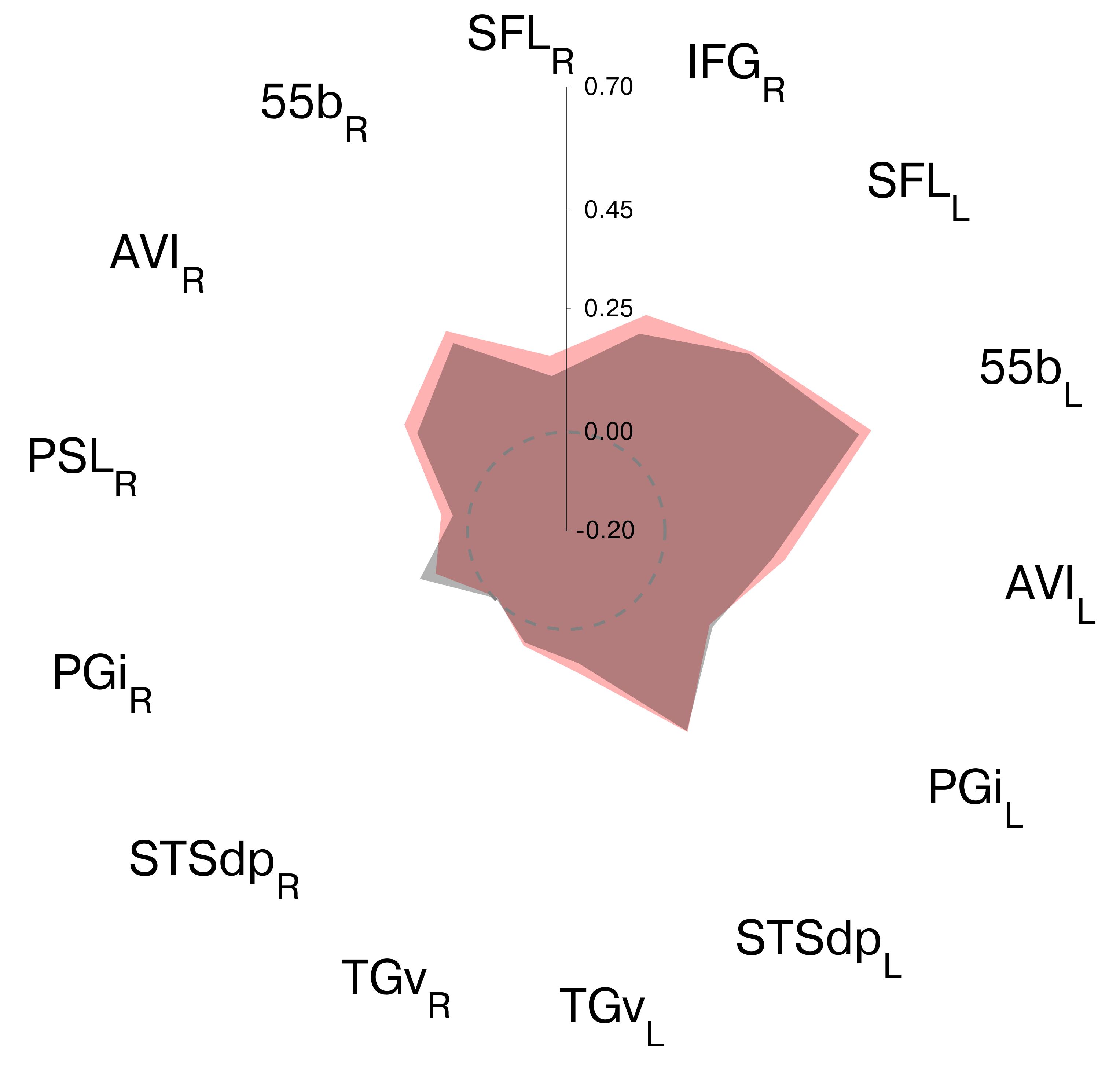

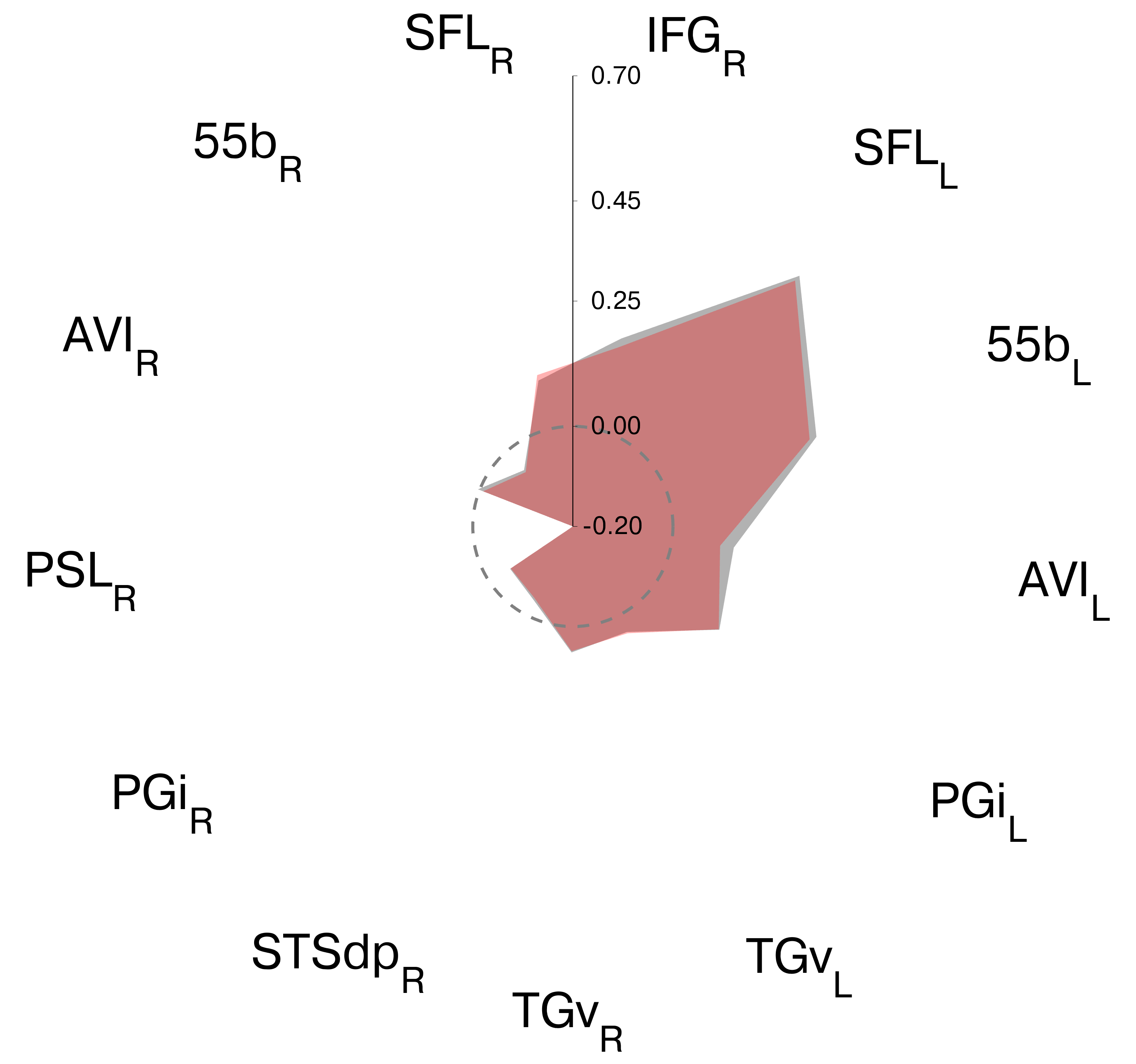

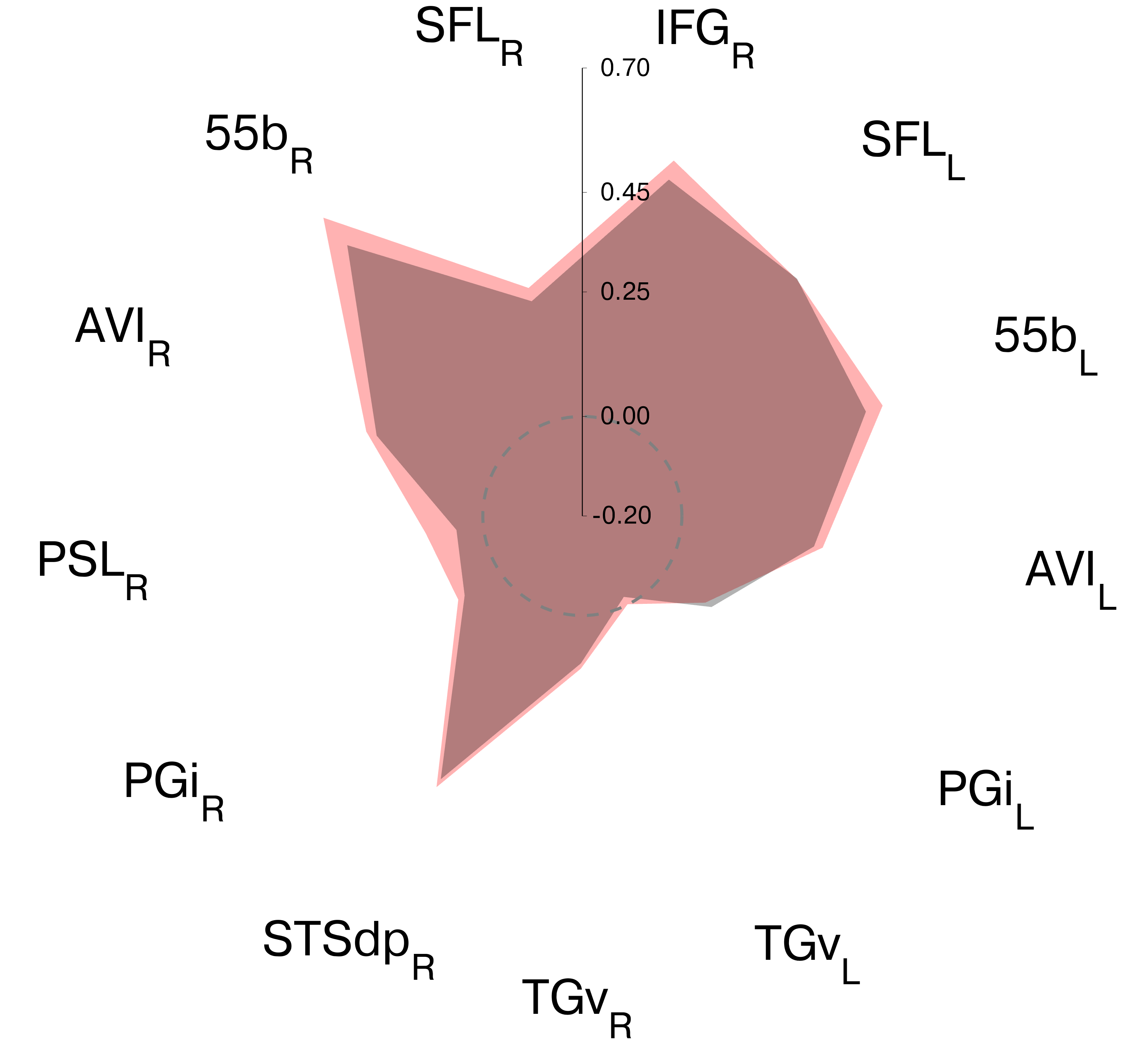

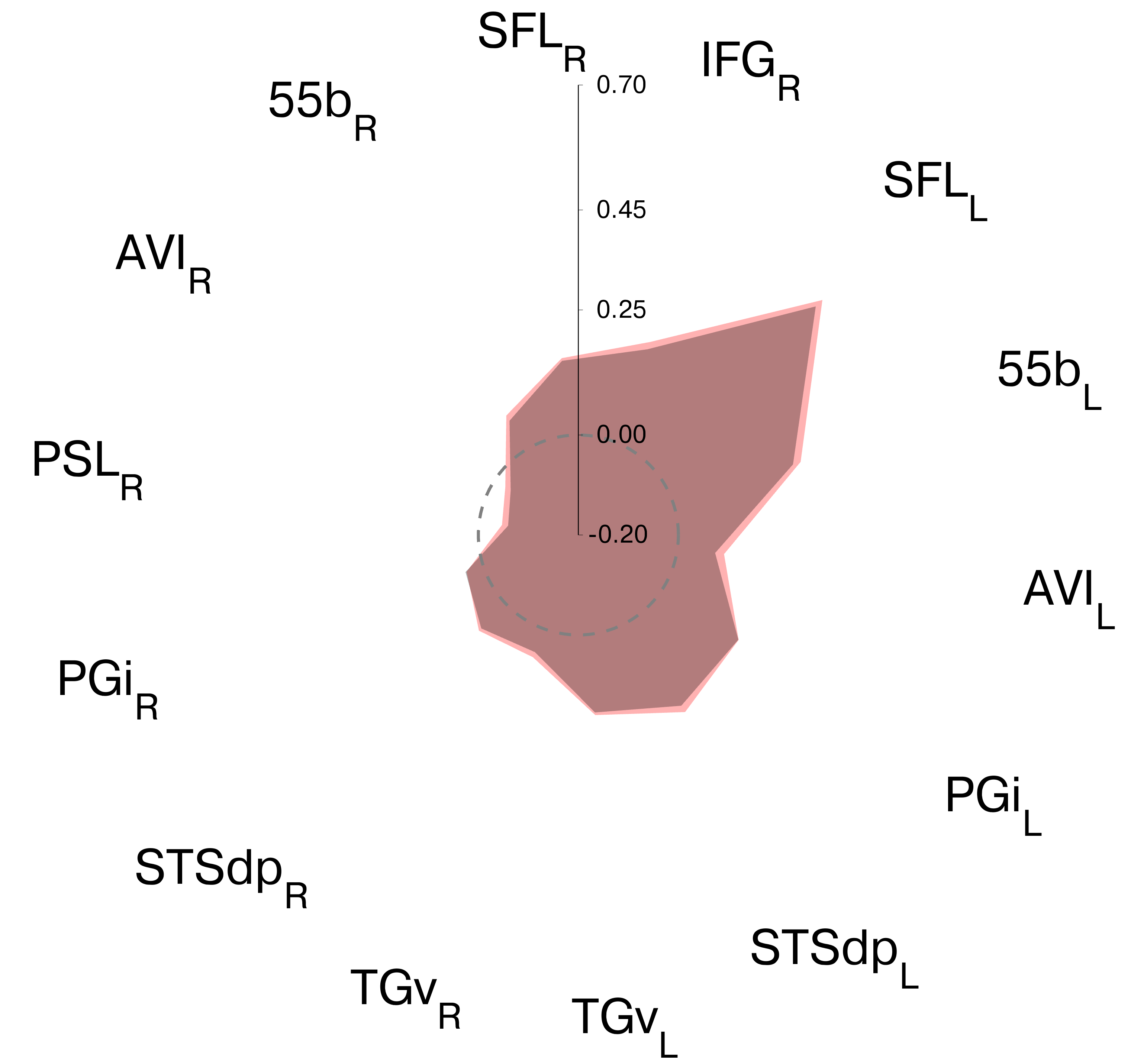


*Pat. A*

*Pat. C*

*Pat. B*

*Pat. E*

*Pat. F*

*Pat. D*


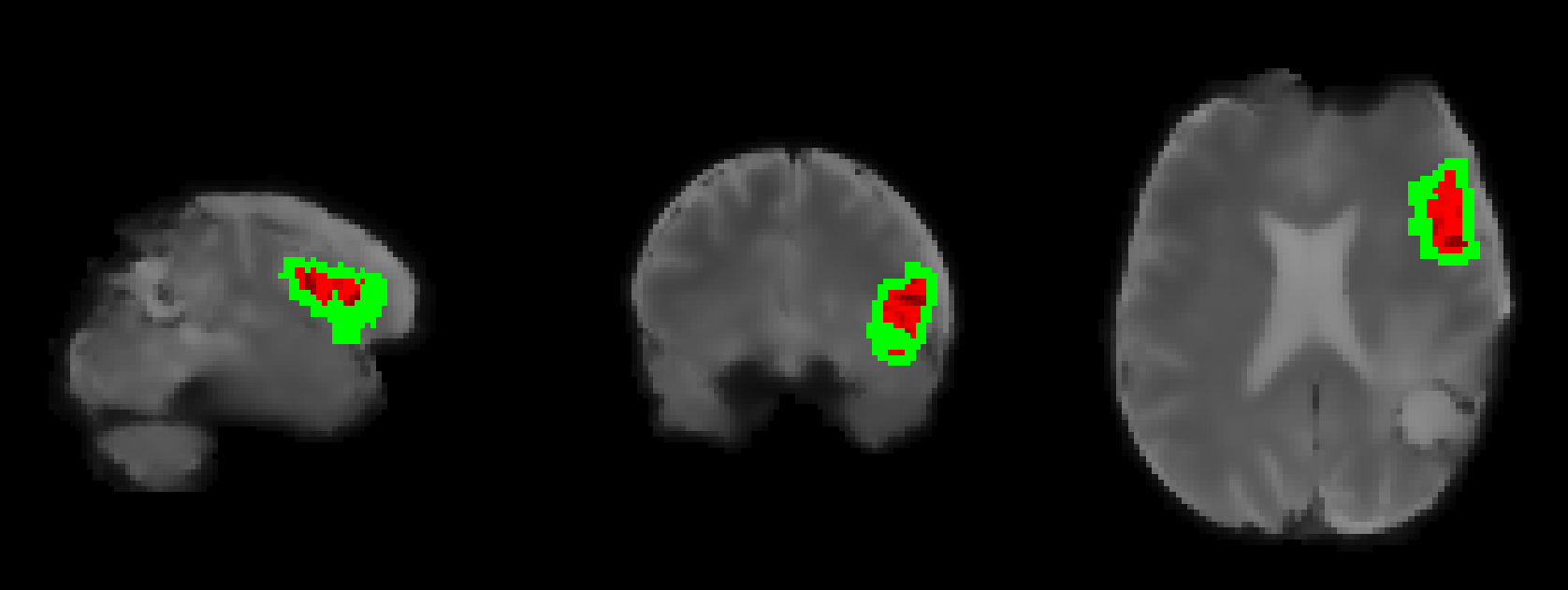


a)

b)

*Supplementary Figure S.5*

*
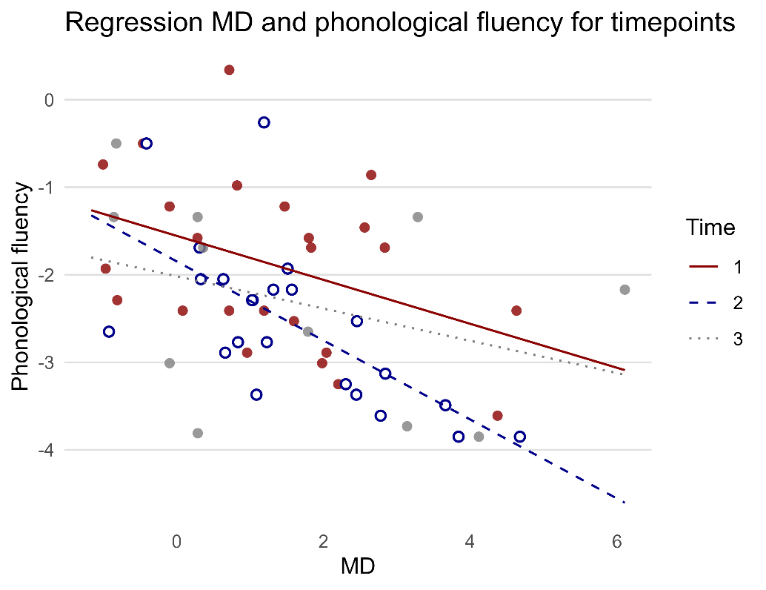
*

**Figure S.5.** Relationship between Manhattan Distance (MD) and phonological fluency across time points. Each dot represents one patient. Colors indicate assessment time points (1 = preoperative, 2 = postoperative, 3 = three-month follow-up), and lines show linear regression fits. Both MD and phonological fluency are expressed as z-scores relative to the control group. Higher MD values indicate greater deviation from the healthy reference network, and lower fluency values indicate poorer phonological fluency. A significant negative association between MD and phonological fluency was observed at the immediate postoperative time point (β = –.67, *p* = .002; age-controlled).

*Supplementary Figure S.6*

*
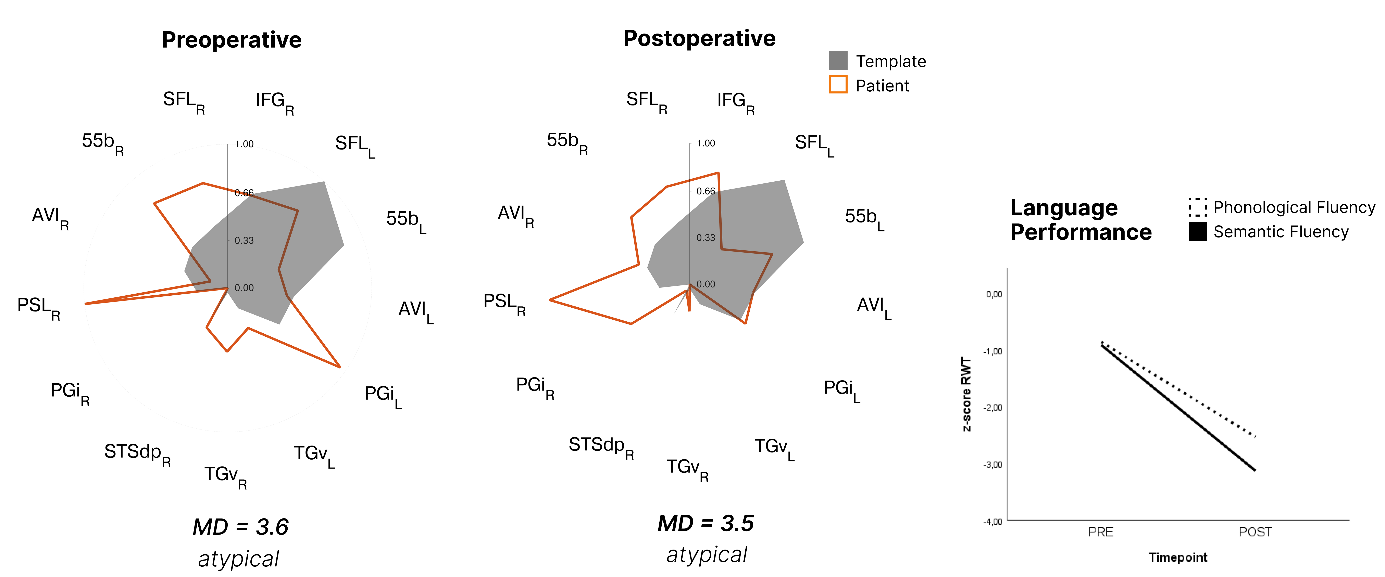
*

**Figure S.6.** Exemplary patient with atypical preoperative (left) and postoperative (middle) fingerprints and corresponding language performance (right). The healthy template is shown in gray and the patient’s fingerprint in orange. While Manhattan distance (MD) values relative to the template are nearly identical at both time points, the fingerprint patterns differ markedly. This illustrates that MD quantifies the degree of deviation but does not capture the specific nature or (mal)adaptive character of the pattern change.
The language performance plot (right) depicts z-scores relative to the control group. This patient exhibited no preoperative deficits, but postoperative declines in both phonological and semantic fluency became evident.

**References**

Glasser, M. F., Coalson, T. S., Robinson, E. C., Hacker, C. D., Harwell, J., Yacoub, E., Ugurbil, K., Andersson, J., Beckmann, C. F., Jenkinson, M., Smith, S. M., & Van Essen, D. C. (2016). A multi-modal parcellation of human cerebral cortex. *Nature*, *536*(7615), 171–178. https://doi.org/10.1038/nature18933

Israel, G. D. (1992). *Determining sample size* (No. PEOD-6). University of Florida, IFAS Extension.

Price, C. J. (2012). A review and synthesis of the first 20 years of PET and fMRI studies of heard speech, spoken language and reading. *NeuroImage*, *62*(2), 816–847. https://doi.org/10.1016/j.neuroimage.2012.04.062

Rolls, E. T., Deco, G., Huang, C.-C., & Feng, J. (2022). The human language effective connectome. *NeuroImage*, *258*, 119352. https://doi.org/10.1016/j.neuroimage.2022.119352
