# Supplementary material for: Identification of abnormal neural language networks by reading “brainprints” in patients with brain tumors
Source: Neuroimage Rep. 2026 Jun 20;6(3):100374. doi: 10.1016/j.ynirp.2026.100374 (PMC13314782; doi:10.1016/j.ynirp.2026.100374)
Supplement: Multimedia component 2 [file mmc2.docx]

**Data and Code Availability Statement**

The data that support the findings of this study are not publicly available due to privacy and ethical restrictions. De-identified data may be available from the corresponding author upon reasonable request and with appropriate institutional and ethical approvals. No custom code was used in this study.
